# Tailoring Nitrogen Terminals on MXene Enables Fast Charging and Stable Cycling Na-Ion Batteries at Low Temperature

**DOI:** 10.1007/s40820-022-00885-7

**Published:** 2022-07-09

**Authors:** Yang Xia, Lanfang Que, Fuda Yu, Liang Deng, Zhenjin Liang, Yunshan Jiang, Meiyan Sun, Lei Zhao, Zhenbo Wang

**Affiliations:** 1 MIIT Key Laboratory of Critical Materials Technology for New Energy Conversion and Storage, State Key Lab of Urban Water Resources and Environment, School of Chemistry and Chemical Engineering, Harbin Institute of Technology, Harbin, 150001 People’s Republic of China; 2 Engineering Research Center of Environment-Friendly Functional Materials, Ministry of Education, Institute of Materials Physical Chemistry, Huaqiao University, Xiamen, 361021 People’s Republic of China; 3 The Institute for Advanced Studies, Wuhan University, Wuhan, 430072 People’s Republic of China; 4 College of Materials Science and Engineering, Shenzhen University, Shenzhen, 518071 People’s Republic of China

**Keywords:** Tailoring nitrogen terminals, Na^+^-solvent co-intercalation, Interfacial kinetics, Fast charging, Low-temperature SIBs

## Abstract

Sodium-ion batteries stand a chance of enabling fast charging ability and long lifespan while operating at low temperature (low-T). However, sluggish kinetics and aggravated dendrites present two major challenges for anodes to achieve the goal at low-T. Herein, we propose an interlayer confined strategy for tailoring nitrogen terminals on Ti_3_C_2_ MXene (Ti_3_C_2_-N_funct_) to address these issues. The introduction of nitrogen terminals endows Ti_3_C_2_-N_funct_ with large interlayer space and charge redistribution, improved conductivity and sufficient adsorption sites for Na^+^, which improves the possibility of Ti_3_C_2_ for accommodating more Na atoms, further enhancing the Na^+^ storage capability of Ti_3_C_2_. As revealed, Ti_3_C_2_-N_funct_ not only possesses a lower Na-ion diffusion energy barrier and charge transfer activation energy, but also exhibits Na^+^-solvent co-intercalation behavior to circumvent a high de-solvation energy barrier at low-T. Besides, the solid electrolyte interface dominated by inorganic compounds is more beneficial for the Na^+^ transfer at the electrode/electrolyte interface. Compared with of the unmodified sample, Ti_3_C_2_-N_funct_ exhibits a twofold capacity (201 mAh g^−1^), fast-charging ability (18 min at 80% capacity retention), and great superiority in cycle life (80.9%@5000 cycles) at − 25 °C. When coupling with Na_3_V_2_(PO_4_)_2_F_3_ cathode, the Ti_3_C_2_-N_funct_//NVPF exhibits high energy density and cycle stability at − 25 °C.

**Supplementary Information:**

The online version contains supplementary material available at 10.1007/s40820-022-00885-7.

## Highlights

- An interlayer confined strategy to realize the substitution of nitrogen terminals between Ti_3_C_2_ MXene layers is proposed.
- The targeted Ti_3_C_2_-N_funct_ anode exhibits fast-charging ability and great superiority in cycle life at − 25 °C.
- Ti_3_C_2_-N_funct_ not only possesses a lower Na-ion diffusion energy barrier and charge transfer activation energy, but also exhibits Na^+^-solvent co-intercalation behavior at low temperature.

**Supplementary Information:**

The online version contains supplementary material available at 10.1007/s40820-022-00885-7.

## Introduction

The ever-increasing demand such as space exploration, military defence, and electric vehicles impels researchers to focus on developing high-performance energy storage devices at low temperature (low-T) [1–3]. Although great efforts have been made to improve the low-T performance, lithium-ion batteries (LIBs) cannot be charged at high currents at low-T and are accompanied by safety risks due to serious dendrite problems [4]. And these issues will be more prominent if commercial graphite is used as the anode. In this regard, researchers turn their attention to sodium-ion batteries (SIBs) owing to the low cost and superior temperature tolerance compared to LIBs [5]. In addition, the de-solvation energy of Na^+^ is about 25–30% smaller than Li^+^, meaning a lower activation barrier of Na (de) insertion [6], which is promising to achieve faster charging and higher battery performance at low-T according to the Arrhenius formula [7]. However, their low-T performance is still limited due to the subzero-temperature induced sluggish ionic transport in electrodes, decreased ions conductivity in electrolytes, and increased impedance between electrode and electrolyte [8, 9].

**Scheme 1:**
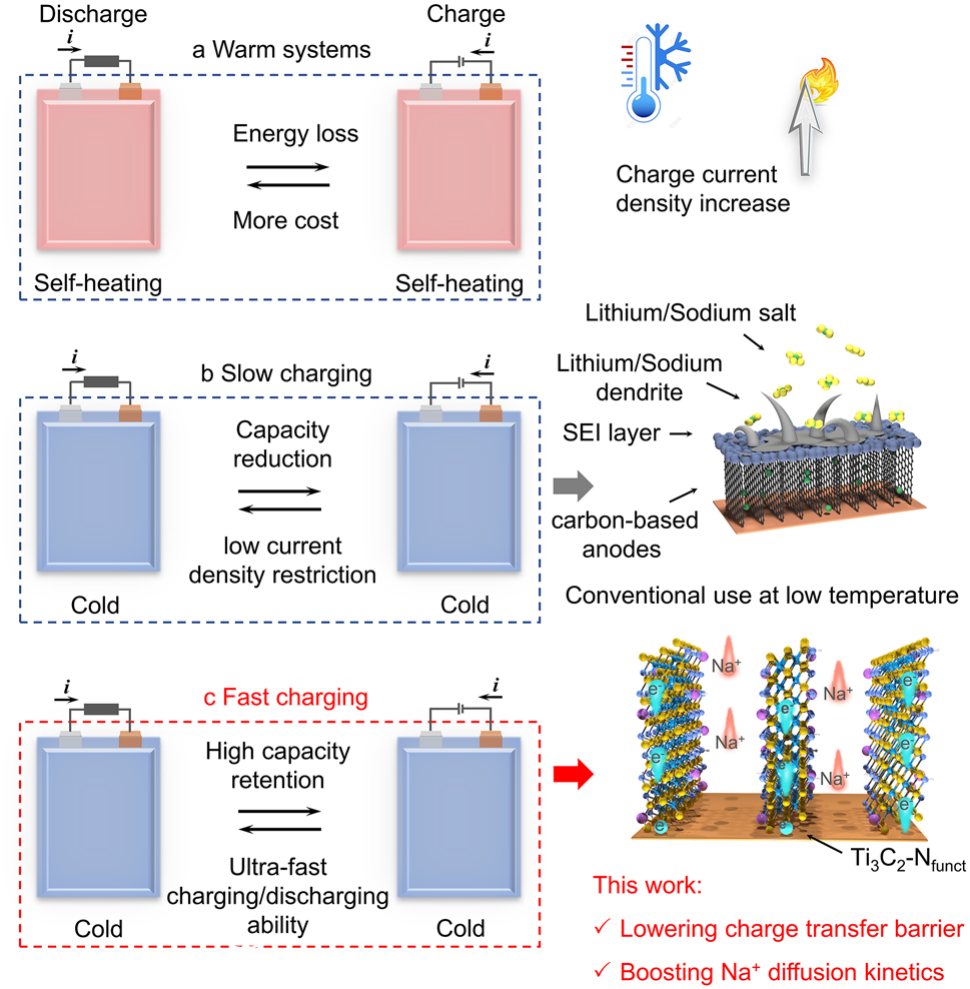
Operational schemes of low-temperature Li/Na-ion batteries and the importance of constructing batteries with fast charging ability at low temperature. **a** Thermal management required during both charge and discharge processes. **b** Slowly charging required or cannot be charged at low temperature. **c** Batteries capable of both fast charging and cycle stability at low temperature

To solve these issues, external warming devices and self-heating systems are applied successively (Scheme 1a), which realize the low-T operation with minimal battery performance loss [10]. However, an extra high current is required for initial activation. Besides, the self-heating devices need to change the structure of the battery and lead to uneven heat distribution; thus there are certain safety risks [11]. Electrolytes are considered as a key reason for battery performance loss, due to the increased viscosity and poor ionic transport at low-T. Great efforts, such as employing low-melting-point solvents, high ions conductive electrolytes, novel salt additives and designing the solvation structure of electrolytes, are made to develop electrolytes applicable to low-T conditions [12–14]. Though several electrolytes (e.g., the mixture of PC and EC, ether-based electrolytes) have been found suitable for low-T operation, low-T batteries are still hindered by the slow charging process (Scheme 1b), if carbon-based anodes are employed [15, 16]. Serious polarization at high current density under low-T leads to the fast arrival of cut-off voltages, resulting in minimal accessible capacity and notorious dendrites formation. As proposed by the Sand time model, the time (*τ*) of the appearance of dendrites is inversely proportional to the square of the current density (*J*) and directly proportional to ionic diffusion (*D*) [17–19].

1 $$ \tau = \pi e^{2} D\left( {1 + \mu_{{\text{c}}} /\mu_{{\text{a}}} } \right)^{2} C_{0}^{2} /4J^{2} $$

in which *μ*_c_ and *μ*_a_ are the mobilities of cation and anion, and *C*_0_ is the initial concentration of the electrolyte. Therefore, it is vital to develop novel anode materials that possess fast interfacial kinetics to conquer these challenges at low-T.

Ti_3_C_2_, a typical member of the MXenes family, possessing high conductivity, large interlayer spacing, low Na^+^ diffusion barrier, high theoretical capacity and appropriate operation voltage, is prospective anode material of SIBs at low-T [20, 21]. However, the surface terminations, such as –OH and –F, highly affect the electronic conductivity and adsorb ability of cations [22, 23]. Restacking of nanosheets causes poor electrolyte wettability and ion accessibility, leading to sluggish ion diffusion and limited reversible capacity [24, 25]. In general, the performance of MXenes has been improved at low-T by constructing 3D architectures to enhance ionic accessibility, interlayer pillaring and surficial groups regulation [9, 26–31]. Tailoring surficial terminals on MXenes is a method to improve the charge transfer process by directly regulating electronic structure [23, 32]. On the other hand, surficial terminals would promote interfacial chemical bonding between ions and substrates to suppress the formation of dendrites [33]. However, compared to the conventional gas doping methods, it is still a great challenge to find a facile and efficient strategy to tailor terminals between Ti_3_C_2_ MXene layers. Moreover, the ions storage mechanism of MXenes under low temperature is still rarely reported.

Herein, to lower the charge transfer barrier and boost ions diffusion kinetics, an interlayer confined strategy for tailoring nitrogen terminals on Ti_3_C_2_ has been proposed, which realizes the fast charging ability at − 25 °C (Scheme 1c). The pre-intercalated cetyltrimethylammonium bromide (CTAB) not only introduces nitrogen source into Ti_3_C_2_ MXene layers for designing nitrogen terminals (Ti_3_C_2_-N_funct_), but also supports the layer structure during the annealing process. The corresponding structural transformation and formation mechanism are demonstrated by variable temperature in situ X-ray diffraction (XRD). As revealed by density functional theory (DFT) calculations, tailoring nitrogen terminals would lead to charge redistribution on Ti_3_C_2_ layer, narrowing the bandgap, endowing the sodiophilicity, and reducing the diffusion energy barrier. The comprehensive X-ray photoelectron spectroscopy (XPS) analyses indicate that the solid electrolyte interface (SEI) formed on the Ti_3_C_2_ and Ti_3_C_2_-N_funct_ electrode shows different composition changes with more inorganic compounds in the interior when formed on Ti_3_C_2_-N_funct_ electrode, which is essential for the Na^+^ transfer at the electrode/electrolyte interface. In addition, the multi-scale physical characterizations show that Ti_3_C_2_-N_funct_ might possess Na^+^-solvent co-intercalation behavior to circumvent the de-solvation process to achieve fast kinetics at low-T. Therefore, the designed Ti_3_C_2_-N_funct_ anodes deliver high reversible capacity, fast-charging ability, and superior cycling stability (80.9% after 5000 cycles) at − 25 °C. When coupling with Na_3_V_2_(PO_4_)_2_F_3_ cathode, the full cells yield high energy density and high capacity retention at − 25 °C.

## Results and Discussion

### Preparation of Ti_3_C_2_-N_funct_ MXene

**Fig. 1:**
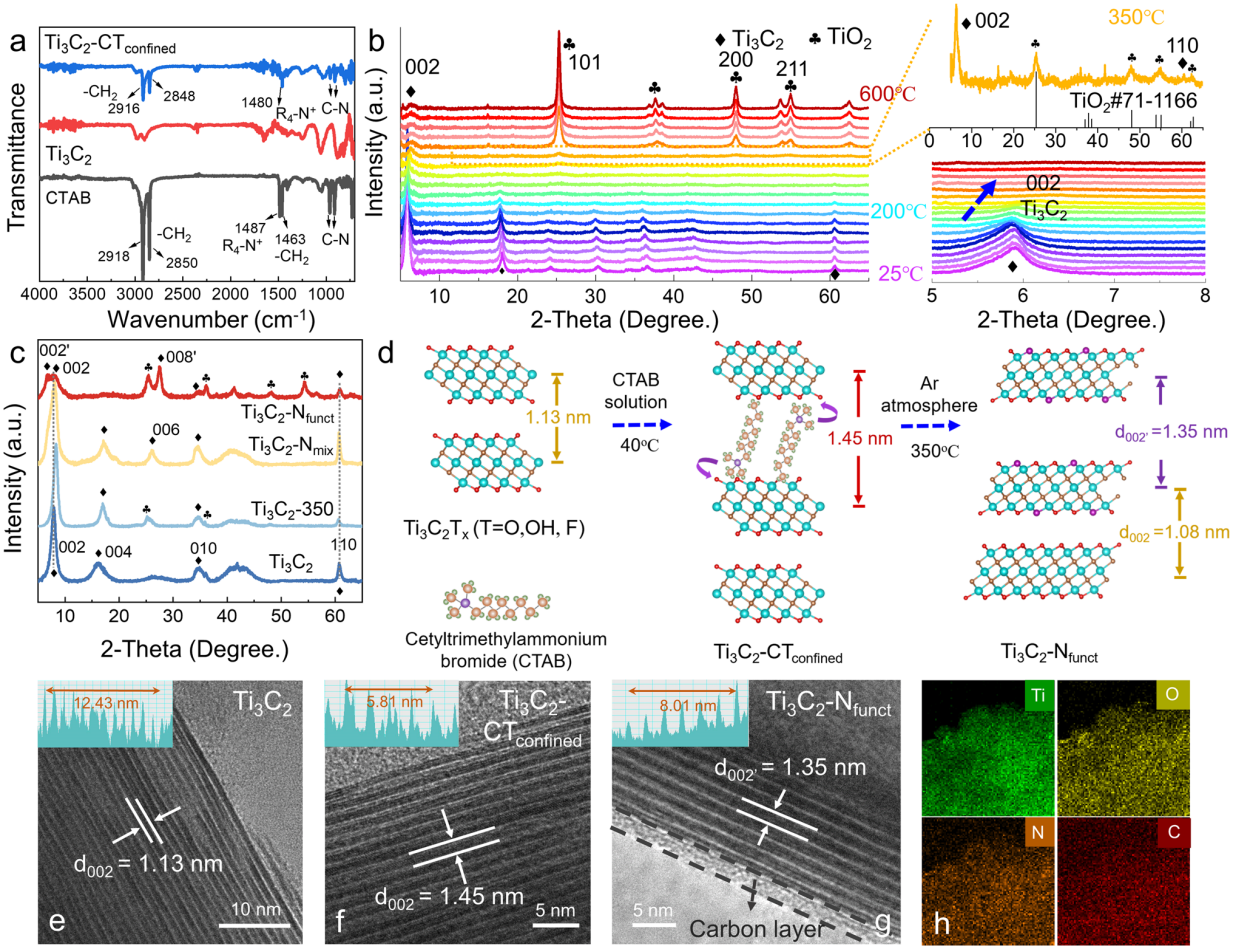
**a** FTIR spectra of CTAB powder, Ti_3_C_2_ and Ti_3_C_2_-CT_confined_. **b** In situ high-temperature XRD patterns, enlarged view within 5°–8° of Ti_3_C_2_-CT_confined_ within 25–600 °C, and enlarged view of XRD pattern at 350 °C. **c** XRD patterns of Ti_3_C_2_, Ti_3_C_2_-350, Ti_3_C_2_-N_mix_ and Ti_3_C_2_-N_funct_. **d** Illustration of Ti_3_C_2_ during the tailoring process. HRTEM images of **e** Ti_3_C_2_, **f** Ti_3_C_2_-CT_confined_ and **g** Ti_3_C_2_-Nfunct. **h** Ti, O, N, and C elements mappings of Ti_3_C_2_-N_funct_

As depicted in Fig. S1, the Ti_3_C_2_-N_funct_ is realized by the in situ thermal decomposition of the pre-intercalated CTAB molecules between the interlayers of Ti_3_C_2_ (Ti_3_C_2_-CT_confined_). First, the intercalation of CTAB and its effect on the layer structure of Ti_3_C_2_ is proved and analysed. The electrostatic interaction between negative-charged surface of Ti_3_C_2_ and the positive-charged CTAB impels Ti_3_C_2_ sheets to expand to accommodate CTAB, which guarantees the intercalation of nitrogen source in layers of Ti_3_C_2_ [34–38]. Fourier transform infrared spectroscopy (FTIR) spectra demonstrate that the peaks corresponding to R_4_N^+^ and -CH_2_ stretching modes of cetyltrimethylammonium (CTA^+^) shift to a lower wave in Ti_3_C_2_-CT_confined_, implying the electrostatic interaction between CTAB and Ti_3_C_2_ (Fig. 1a). XRD patterns exhibit that the (002) peak of Ti_3_C_2_-CT_confined_ become wider and shift to a lower angle compared to Ti_3_C_2_ (Fig. S2). This is completely different from the simple overlaying of (002) peak and CTAB peak observed in Ti_3_C_2_-CT_mix_ (Ti_3_C_2_ physically mixed with CTAB powders), further verifying that CTAB has successfully intercalated in the layers of Ti_3_C_2_. Such a phenomenon is consistent with high-resolution transmission electron microscopy (HRTEM) images, wherein the interlayer spacing of Ti_3_C_2_ enlarges to 1.45 nm from 1.13 nm after CTAB pre-intercalation (Fig. 1e, f). Second, the interlayered confined CTAB will decompose and part of the released N atoms will be captured by Ti_3_C_2_ to form nitrogen terminals during the annealing process. Temperature is a crucial factor that strongly influence the formation of surface terminations on MXenes. To determine the optimal tailoring temperature, in situ high-temperature XRD and thermogravimetric analysis (TGA) were performed to track the thermal structural transformation of Ti_3_C_2_-CT_confined_ as demonstrated in Figs. 1b and S3. When the temperature rises from 25 to 200 °C, the (002) peak of Ti_3_C_2_-CT_confined_ slightly shifts to a higher angle, and a weight loss of 2.2% is observed both in Ti_3_C_2_ and Ti_3_C_2_-CT_confined_, which could be ascribed to the loss of water adsorbed in the layers of Ti_3_C_2_ [39]. The salient deviation of TGA curves from 200 to 250 °C between Ti_3_C_2_ and Ti_3_C_2_-CT_confined_ can be attributed to the decomposition of CTAB, in which N atoms are released from CTAB (Fig. S3). With the temperature increasing above 300 °C, the removal of surficial terminals will take place as in previous reports [29, 40]. When the temperature rises to 350 °C, there is an obvious drop in the TGA curves of Ti_3_C_2_-CT_confined_ compared to Ti_3_C_2_ and pure CTAB powder. Such a phenomenon could be attributed to the low carbonization ratio of CTAB and the confined effect of MXene on CTAB. A new peak is gradually formed at 25.2° (Fig. 1b), which belongs to anatase TiO_2_ (JCPDS No. 71-1166) [41]. However, the (002) and (110) peaks belonging to Ti_3_C_2_ are still maintained, implying the layer structure could be kept at 350 °C. When temperature keeps rising, the intensity of (002) peak decreases and more characteristic peaks of anatase TiO_2_ appear, confirming that Ti_3_C_2_ would inevitably transform to anatase TiO_2_ at a higher temperature. To confirm the tailoring temperature, the electrochemical performance of Ti_3_C_2_-CT_confined_ annealing between 300 and 400 °C were compared, as shown in Fig. S4. Due to the low tailoring efficiency under 300 °C and serious oxidation of Ti_3_C_2_ under 400 °C, 350 °C is considered as an appropriate temperature for tailoring nitrogen terminals on Ti_3_C_2_.

To illustrate the important role of CTAB pre-intercalation in the preparation of Ti_3_C_2_-N_funct_, XRD patterns of Ti_3_C_2_-N_funct_, Ti_3_C_2_-N_mix_ (product of Ti_3_C_2_-CT_mix_ after annealing at 350 °C), Ti_3_C_2_-350 (Ti_3_C_2_ powder after annealing at 350 °C), and Ti_3_C_2_ are compared (Fig. 1c). The main diffraction peaks of Ti_3_C_2_-N_mix_ are similar to that of Ti_3_C_2_ and the peak of anatase TiO_2_ is also observed in Ti_3_C_2_-350. Moreover, a thick carbon layer on Ti_3_C_2_-N_mix_ also indicated that CTAB decomposed outside of Ti_3_C_2_ layers (Fig. S5). Since the introduction of CTAB, more TiO_2_ peaks are observed in Ti_3_C_2_-N_funct_ than in other samples. The split-up of (002) peak in Ti_3_C_2_-N_funct_ further indicates that Ti_3_C_2_-N_funct_ still keeps large interlayer spacing after the decomposition of pillared CTAB. This is consistent well with the TEM images shown in Fig. 1e–g, wherein Ti_3_C_2_ shows a layer distance of 1.13 nm and expands to 1.45 nm after the CTAB intercalation, then reduces to 1.35 nm after thermal treatment. Figure 1d illustrates the structure evolution of Ti_3_C_2_ during the preparation of Ti_3_C_2_-N_funct_ and the phenomenon of (002) peak splitting. Moreover, the N element is distributed uniformly on Ti_3_C_2_-N_funct_ (Fig. 1h). The crinkled nanosheets remain after thermal treatment (Fig. S6), ensuring electrolyte wettability and ion accessibility. These results demonstrate that CTAB pre-intercalation and thermal treatment can achieve uniform N doping and make Ti_3_C_2_-N_funct_ maintain well-layered structure with a large lattice distance, while the physical mixing of CTAB cannot achieve this effect.

### Electronic Structure Analysis

**Fig. 2:**
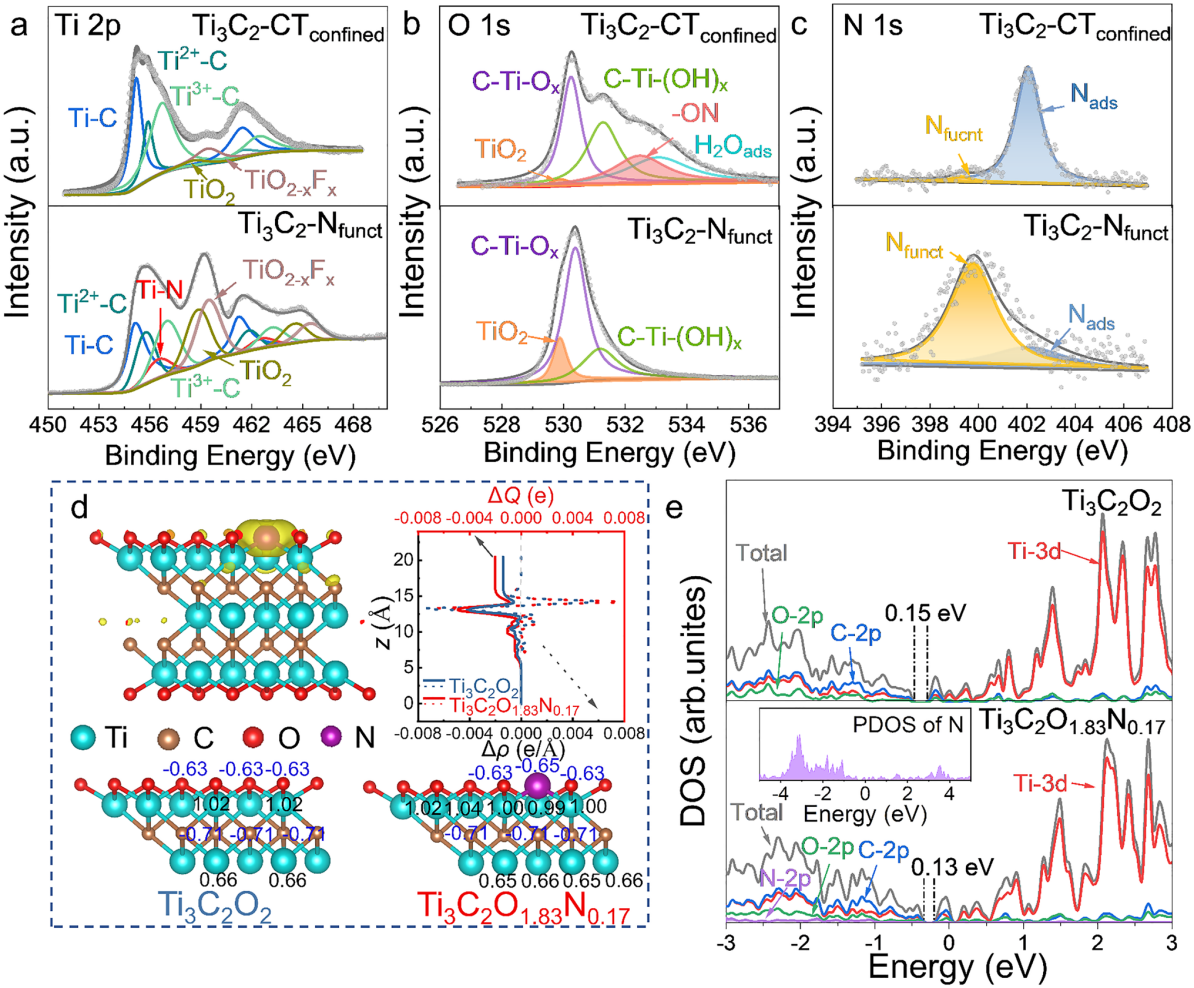
High-resolution XPS spectra of **a** Ti 2*p*, **b** O 1*s*, and **c** N 1*s* of Ti_3_C_2_-CT_confined_ and Ti_3_C_2_-N_funct_. **d** Electron density difference (EDD) of Ti_3_C_2_O_1.83_N_0.17_, in which yellow is positive; the line-profiles of plane-averaged EDD Δ*ρ* (dot line) and amount of transferred charge Δ*Q* (solid line); the atomic populations of Ti_3_C_2_O_2_ and Ti_3_C_2_O_1.83_N_0.17_. **e** The calculated density of states (DOS) and partial density of states (PDOS) of Ti_3_C_2_O_2_ and Ti_3_C_2_O_1.83_N_0.17_

XPS was applied to record the difference of the valence states of Ti/O elements and the existence of N between Ti_3_C_2_-CT_confined_ and Ti_3_C_2_-N_funct_. The Ti 2*p* spectra of Ti_3_C_2_-CT_confined_, Ti_3_C_2_-N_funct_, and Ti_3_C_2_ are provided in Figs. 2a and S7a, a new peak appears at 456.5 eV, which is attributed to Ti-N in Ti_3_C_2_-N_funct_, indicating that N atoms bond with Ti atoms on Ti_3_C_2_ [42–44]. Moreover, the O 1*s* spectra reflect the change of surficial composition on Ti_3_C_2_ (Fig. 2b) [45, 46]. The peak at 533.8 eV, corresponding to -ON derived from the electrostatic interaction between N atoms in CTAB and surficial O-terminals of Ti_3_C_2_, disappears in Ti_3_C_2_-N_funct_. In addition, the C-Ti peak of Ti_3_C_2_, Ti_3_C_2_-CT_confined_ and Ti_3_C_2_-N_funct_ also reflect the electrostatic interaction between CTAB and Ti_3_C_2_ (Fig. S7). Different from the existence of lattice substitution for C atoms (396.0 eV) in previous reports, the N 1*s* peak can only be split into two peaks located at 399.8 and 402.0 eV in this work, corresponding to the surficial N-terminals and adsorbed N atoms, labelled as N_funct_ and N_ads_ (Fig. 2c) [32, 42]. It is clear that, after the thermal treatment, the proportion of N_funct_ increases sharply, further verifying the successful transformation of adsorbed N atoms in CTAB to the bonding N atoms in Ti_3_C_2_-N_funct_. The concentrations of nitrogen element in Ti_3_C_2_-CT_confined_ and Ti_3_C_2_-N_funct_ are 3.63 and 1.05 at%, as exhibited in Table S1.

DFT calculation was conducted to figure out the effect of tailoring N-terminals on the electronic structure of Ti_3_C_2_. A 3 × 2 × 1 supercell of Ti_3_C_2_O_2_ is constructed as the initial structure as demonstrated in Fig. S8a. An oxygen atom is substituted by a nitrogen atom in the supercell to approximate the N atoms concentration in Ti_3_C_2_-N_funct_ (Fig. S8b), corresponding to a chemical stoichiometry of Ti_3_C_2_O_1.83_N_0.17_. Charge accumulation around N atoms and depletion near Ti atoms are verified by electron density difference (EDD) in Fig. 2d. Based on Eq. 2, the plane-averaged EDD Δ*ρ* and the charge transfer Δ*Q* are delivered to quantitatively compare the charge discrepancy after N substitution (Fig. 2d right) [8]. It could be observed that more charge will transfer from N atoms to Ti atoms compared to that of O atoms. The Mulliken charge distribution demonstrates that Ti atoms bond with N atoms and inner Ti atoms obtain more electrons with a reduced charge (Fig. 2d). These results are consistent with Ti 2*p* XPS spectra in Fig. 2a, in which the peaks of Ti-C and Ti^2+^-C slightly shift to low binding energy due to more electrons provided by N atoms. The above observations suggest that the N- terminals could induce a charge redistribution, leading to the formation of more active sites of redox reactions [44].

2 $$ \Delta Q\left( z \right) = \smallint \Delta \rho \left( {z^{\prime } } \right){\text{d}}z^{\prime } $$

The density of states (DOS) of Ti_3_C_2_O_2_ and Ti_3_C_2_O_1.83_N_0.17_ are shown in Fig. 2e. As observed, the conduction band (CB) of Ti_3_C_2_O_2_ and Ti_3_C_2_O_1.83_N_0.17_ is mainly contributed by Ti 3*d*, while the valence band (VB) originates from the hybridization of Ti 3*d*, C 2*p* and O 2*p* [32]. The bandgap is reduced from 0.15 to 0.13 eV after N atom substitution, manifesting that electrons could easily migrate from VB to CB, implying the enhanced electronic conductivity of Ti_3_C_2_O_1.83_N_0.17_. The DOS shape of Ti_3_C_2_O_1.83_N_0.17_ below the Fermi level is sharper, indicating that the substitution of N atoms makes electrons more localized [47], which is beneficial for the chemical bonding with Na^+^ to increase the driving force for nucleation to inhibit the formation of metallic Na nuclei [33]. Such improvement is particularly important at low temperatures and can effectively inhibit the formation of sodium dendrites to achieve fast charging ability. This speculation will be confirmed in the next part.

Furthermore, the adsorption energy of Na atom on Ti_3_C_2_O_2_ and Ti_3_C_2_O_1.83_N_0.17_ is investigated to demonstrate the sodiophilicity of N-groups on Ti_3_C_2_. The optimized geometric structures of a Na atom on monolayer Ti_3_C_2_O_2_ and Ti_3_C_2_O_1.83_N_0.17_ are exhibited in Figs. S9 and S10. As exhibited in Fig. S11, after tailoring N-terminals on Ti_3_C_2_, the adsorption energy becomes more negative, confirming the adsorption stability of Na. It is worthy to notice that the adsorption sites near the N atoms possess the lowest adsorption energy among types of C top and Ti top, suggesting that the surficial N-terminals would induce Na^+^ to form a Na–N–Ti interaction to keep the lowest energy state. Such a phenomenon indicates that the existence of N-terminal on Ti_3_C_2_ could regulate the Na deposition behavior. Moreover, the optimized structures of a Na atom intercalating in double layers of Ti_3_C_2_O_2_ and Ti_3_C_2_O_1.83_N_0.17_ are exhibited in Fig. S12, wherein the Ti_3_C_2_O_1.83_N_0.17_ possesses larger interlayer spacing, in accordance with HRTEM results (Fig. 1 g). The negative formation energy suggests that Na^+^ prefers to intercalate into the layers of Ti_3_C_2_. The result suggests that the surficial nitrogen terminals regulation could effectively improve the electronic conductivity, enhancing the sodiophilicity of Ti_3_C_2_, and improve the possibility of Ti_3_C_2_ for accommodating more Na atoms, further enhancing the Na^+^ storage capability of Ti_3_C_2_.

### Electrochemical Performance at − 25 °C

**Fig. 3:**
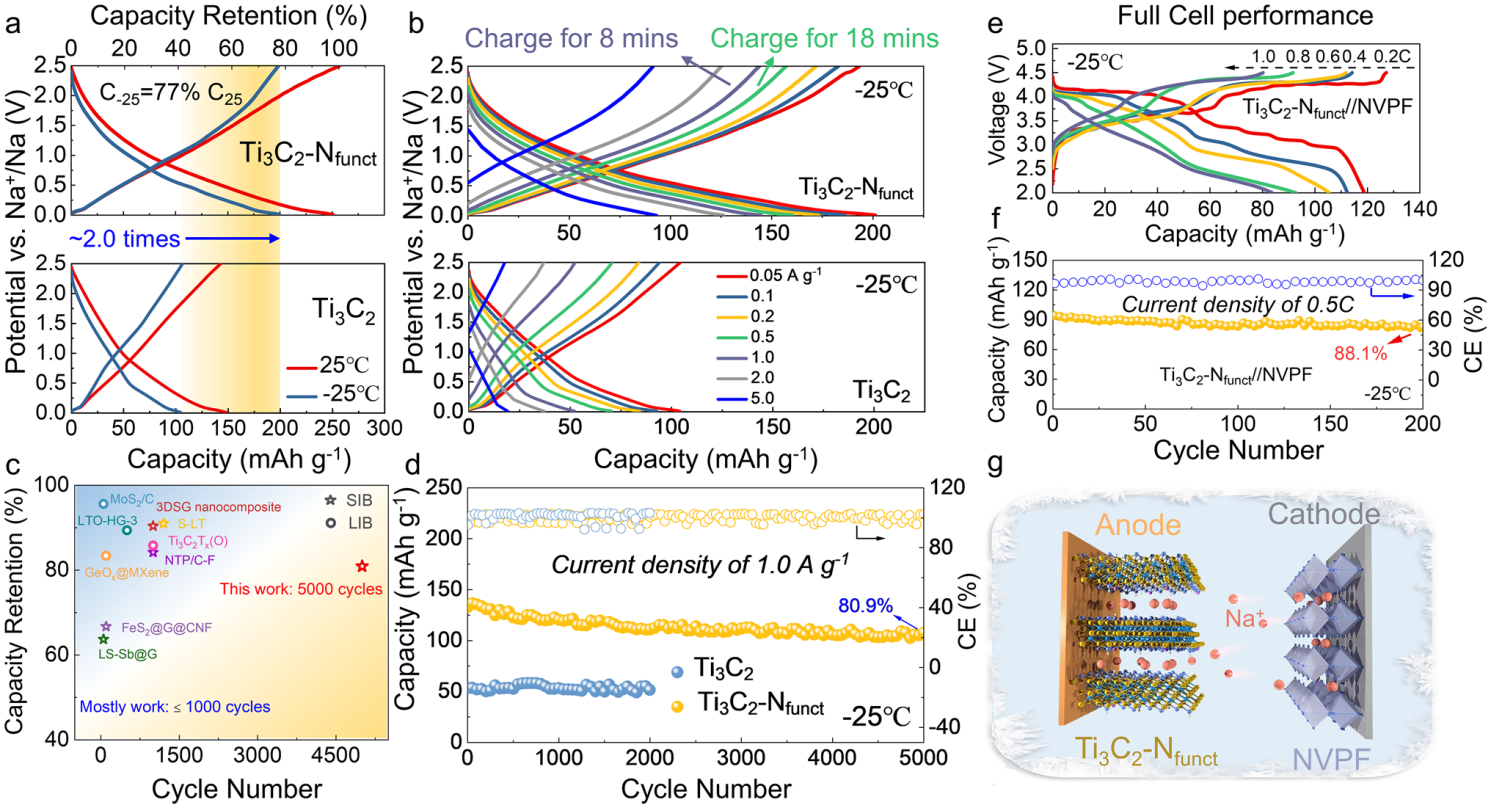
Electrochemical performance of Ti_3_C_2_ and Ti_3_C_2_-N_funct_ anodes in Na-ion half-cells and Ti_3_C_2_-N_funct_//NVPF full-cell. **a** The second charge/discharge curves of Ti_3_C_2_ and Ti_3_C_2_-N_funct_ anodes in Na-ion half-cells at 0.05 A g^−1^ under 25 and − 25 °C. **b** Rate capabilities of half-cells at − 25 °C. **c** Comparison of low-T cycle performance of Ti_3_C_2_-N_funct_ vs. other reported LIBs and SIBs. **d** Cycle performance of half cell at − 25 °C. **e** Rate performance (1C represents 0.128 mA g^−1^) and **f** cycling stability at 0.5C of Ti_3_C_2_-N_funct_//NVPF full-cell. **g** Illustration of Ti_3_C_2_-N_funct_//NVPF full-cell

Based on the well-tailored Ti_3_C_2_-N_funct_ anode, half-cells are assembled with metallic Na to evaluate the electrochemical performance at − 25 °C. Ti_3_C_2_-N_funct_ delivers a much higher capacity, which is nearly 2 times that of Ti_3_C_2_ at 25 and − 25 °C (Fig. 3a). Besides, the capacity loss caused by the temperature drop is greatly reduced, and the reversible capacity of Ti_3_C_2_-N_funct_ at − 25 °C is about 77% of room temperature. The first charging/discharging curves of Ti_3_C_2_ and Ti_3_C_2_-N_funct_ demonstrate that SEI forms in the first discharge process, which leads to the unpleasant initial Coulombic efficiency (Fig. S13). Figure 3b is the rate capability of the two anodes at − 25 °C, the specific capacities of Ti_3_C_2_-N_funct_ are 201, 182, 172, 160, 143, 126, and 90 mAh g^−1^ at 0.05, 0.1, 0.2, 0.5, 1.0, 2.0, and 5.0 A g^−1^, while about half of the capacity is obtained in Ti_3_C_2_. The Ti_3_C_2_-N_funct_ anode delivers fast-charging/discharging ability at low-T, which could recharge to 80% capacity (160 mAh g^−1^) within 18 min and 72% capacity (144 mAh g^−1^) in 8 min (Fig. S14). After 5000 cycles at 1.0 A g^−1^ at − 25 °C, the Ti_3_C_2_-N_funct_ electrode maintains capacity retention of 80.9% (Fig. 3d), superior to most reported low-T batteries as shown in Table S2 and Fig. 3c [48–50].

In addition, Na_3_V_2_(PO_4_)_2_F_3_ cathode, due to its high capacity retention and reversibility of Na^+^ at low-T [51], was selected as the basis of eventual full-cell construction (Fig. 3g). The electrochemical performance of Na_3_V_2_(PO_4_)_2_F_3_ cathodes are shown in Fig. S15. The reversible capacities of 118 mAh g^−1^ (based on the mass of cathode material) at 0.2C have been achieved (1C represents 0.128 mA g^−1^), which keeps high capacity retention (95%) compared to room temperature (Figs. 3e and S16). The designed Ti_3_C_2_-N_funct_//NVPF SIB displays excellent cycle stability at − 25 °C (Fig. 3f), which keeps capacity retention of 88.1% after 200 cycles at 0.5C. Moreover, as shown in Fig. S17, the Ti_3_C_2_-N_funct_//NVPF full cell achieves a maximum energy density and power density of 414.14 Wh kg^−1^ and 763.98 W kg^−1^ at − 25 °C, respectively (based on the cathode material). Hence, the interlayer confined strategy for tailoring N-terminals on Ti_3_C_2_ is effective for improving the Na^+^ storage performance at low-T of MXenes including temperature adaptability, fast-charging ability, and ultra-long lifespan.

### Analysis of Kinetics of Ti_3_C_2_-N_funct_ Anode at Low Temperature

**Fig. 4:**
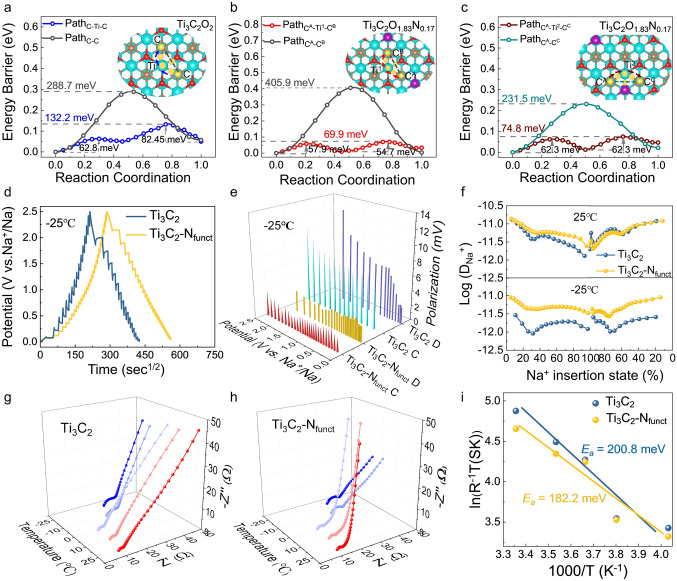
Kinetics analysis: the diffusion energy barrier of Na^+^ along different paths in **a** Ti_3_C_2_O_2_ and **b**-**c** Ti_3_C_2_O_1.83_N_0.17_. **d** Charge–discharge GITT curves of Ti_3_C_2_ and Ti_3_C_2_-N_funct_. **e** Comparison of polarization of the two electrodes at − 25 °C during the charge/discharge process. **f** The Na^+^ diffusion coefficients of Ti_3_C_2_ and Ti_3_C_2_-N_funct_ at 25 °C and − 25 °C. Temperature-dependent EIS study of **g** Ti_3_C_2_ and **h** Ti_3_C_2_-N_funct_ anodes from − 25 to 25 °C. **i** Arrhenius plot of the resistance contributions of the charge transfer resistance (*R*_ct_) with the derived activation energies (*E*_a_) for the two electrodes

Given a deeper insight into the effects of tailoring N-terminals on Ti_3_C_2_, DFT calculations and various experimental analyses were delivered to investigate the kinetics of Ti_3_C_2_ and Ti_3_C_2_-N_funct_. To clarify the fast diffusion process of Na^+^ in Ti_3_C_2_-N_funct_, DFT calculations of energy barrier of possible Na^+^ diffusion paths in bilayer framework for Ti_3_C_2_O_2_ and Ti_3_C_2_O_1.83_N_0.17_ were carried out. As previous results in Fig. S11, the site on top of C atoms is preferred Na adsorption sites. Therefore, two possible pathways of Na^+^ between the two nearest neighboring C sites in the bilayer of Ti_3_C_2_O_2_ are explored (Fig. 4a). For O-Path_C-C_, the Na^+^ is hopping directly to the nearest C site in a one-step path; for O-Path_C-Ti-C_, the Na^+^ is migrating along the pathway from the top of C atoms to the top of Ti atoms and then to the nearest C atoms [22, 52]. The calculated energy barrier of two paths are 288.7 and 132.2 meV, indicating that Na^+^ tends to migrate along the path_C-Ti-C_. When an oxygen atom is substituted by a nitrogen atom in Ti_3_C_2_O_1.83_N_0.17_, C atoms with different chemical environment are emerging, labelled as C^A^, C^B^, C^C^, and C^D^. Therefore, three diffusion directions of Na^+^ in the bilayer of Ti_3_C_2_O_1.83_N_0.17_ are explored as shown in Figs. 4b, c and S18–S19. It is noteworthy that the diffusion energy barrier of path_C-Ti-C_ in Ti_3_C_2_O_1.83_N_0.17_ is lower than that of Ti_3_C_2_O_2_ and the energy barrier for Na^+^ hopping over the Ti-N bonds is lower than that of Ti–O bonds, demonstrating that tailoring N-terminals on Ti_3_C_2_ is efficient to facilitate Na^+^ diffusion. Especially, the N-Path_C_^A^_-Ti_^1^_-C_^B^ for Na^+^ migration is presented in Fig. 4b, with a calculated energy barrier as low as 69.9 meV, which is much lower than that of typical Na-ion insertion materials as previous reported, such as TiO_2_ (2.20 eV), TiS_2_ (1.20 eV), and TiSe_2_ (0.5 eV), confirming the fast kinetics of Ti_3_C_2_-N_funct_ [53–55].

The Na^+^ storage behavior of Ti_3_C_2_-N_funct_ electrodes at − 25 °C is verified by the cyclic voltammetry (CV) curves at various scan rates from 0.2 to 2.0 mV s^−1^ (Fig. S20a). According to Eq. 3:

3 $$ i = av^{b} $$

in which the Na^+^ storage mechanisms can be defined as faradic ion intercalation (*b* = 0.5) or capacitive response (*b* = 1.0) [56]. The calculated *b* values for peak C_1_, C_2_, A_1_, and A_2_ are 0.89, 0.95, 0.88, and 0.94, respectively, demonstrating that the Ti_3_C_2_-N_funct_ electrodes possess fast kinetics at − 25 °C (Fig. S20b). The Na^+^ diffusion coefficients and polarization of Ti_3_C_2_ and Ti_3_C_2_-N_funct_ electrodes at − 25 °C were identified by the galvanostatic intermittent titration technique (GITT) (Fig. 4d, details in Fig. S21). The Ti_3_C_2_-N_funct_ delivers much lower polarization during the charge/discharge process than that of Ti_3_C_2_ at − 25 °C, which might suppress the nucleation rate of Na dendrites as shown in Fig. 4e [18]. The calculated Na^+^ diffusion coefficients of Ti_3_C_2_ and Ti_3_C_2_-N_funct_ electrodes at 25 and − 25 °C are demonstrated in Fig. 4f. At room temperature, the Na^+^ diffusion coefficients of Ti_3_C_2_ and Ti_3_C_2_-N_funct_ are very close. However, while transferred to − 25 °C, Ti_3_C_2_-N_funct_ could nearly maintain the same Na^+^ diffusion coefficients at room temperature, which is much higher than that of Ti_3_C_2_, indicating the better adaptability of Ti_3_C_2_-N_funct_ at low-T. This discrepancy is significant, as proposed by the Sand time model [17], the nearly 5.6 times difference in Na^+^ diffusion coefficients is expected to prolong the time (*τ*) of dendrites appearing, which could guarantee the cycle stability at low-T. Therefore, beneficial from the interfacial chemical bonding of Na^+^ and the low nucleation rate of Na dendrites, the Ti_3_C_2_-N_funct_ delivers excellent cycle stability.

The interfacial charge transfer dominates battery performance at low-T as previous studies reported, the activation energy (*E*_a_) holds the key to determining the interfacial kinetics [7]. Based on the Butler–Volmer equation and Arrhenius equation, the relationship between charge transfer resistance (*R*_ct_) and *E*_a_ is generalized as:

4 $$ T/R_{{{\text{ct}}}} = A\exp \, \left( { - E_{{\text{a}}} /RT} \right) $$

in which *A* is a constant, *R* is the gas constant, *T* is the temperature. As shown in Fig. 4g-h, the temperature-dependent EIS was performed to measure the value of *R*_ct_ from − 25 to 25 °C for Ti_3_C_2_ and Ti_3_C_2_-N_funct_ electrodes. The equivalent circuit model of Ti_3_C_2_ and Ti_3_C_2_-N_funct_ electrodes is exhibited in Fig. S22. The fitted *R*_ct_ values of Ti_3_C_2_ and Ti_3_C_2_-N_funct_ electrodes at various temperatures are exhibited in Table S3. It is noteworthy that the *R*_ct_ values of two electrodes are low, indicating the fast charge transfer process at − 25 °C. Based on Eq. 4, the calculated values of *E*_a_ for Ti_3_C_2_ and Ti_3_C_2_-N_funct_ electrodes are 200.8 and 182.2 meV (Fig. 4i), respectively. This result demonstrates that Ti_3_C_2_-N_funct_ could serve a nearly 10% reduction in activation energy during the interfacial charge transfer process than that of Ti_3_C_2_. All these observations further confirm that tailoring N-terminals on Ti_3_C_2_ not only accelerate interfacial kinetics and Na^+^ diffusion but also lower the charge transfer energy barrier, which is essential for achieving fast-charging ability at low-T.

### Analysis of Electrode/Electrolyte Interface

**Fig. 5:**
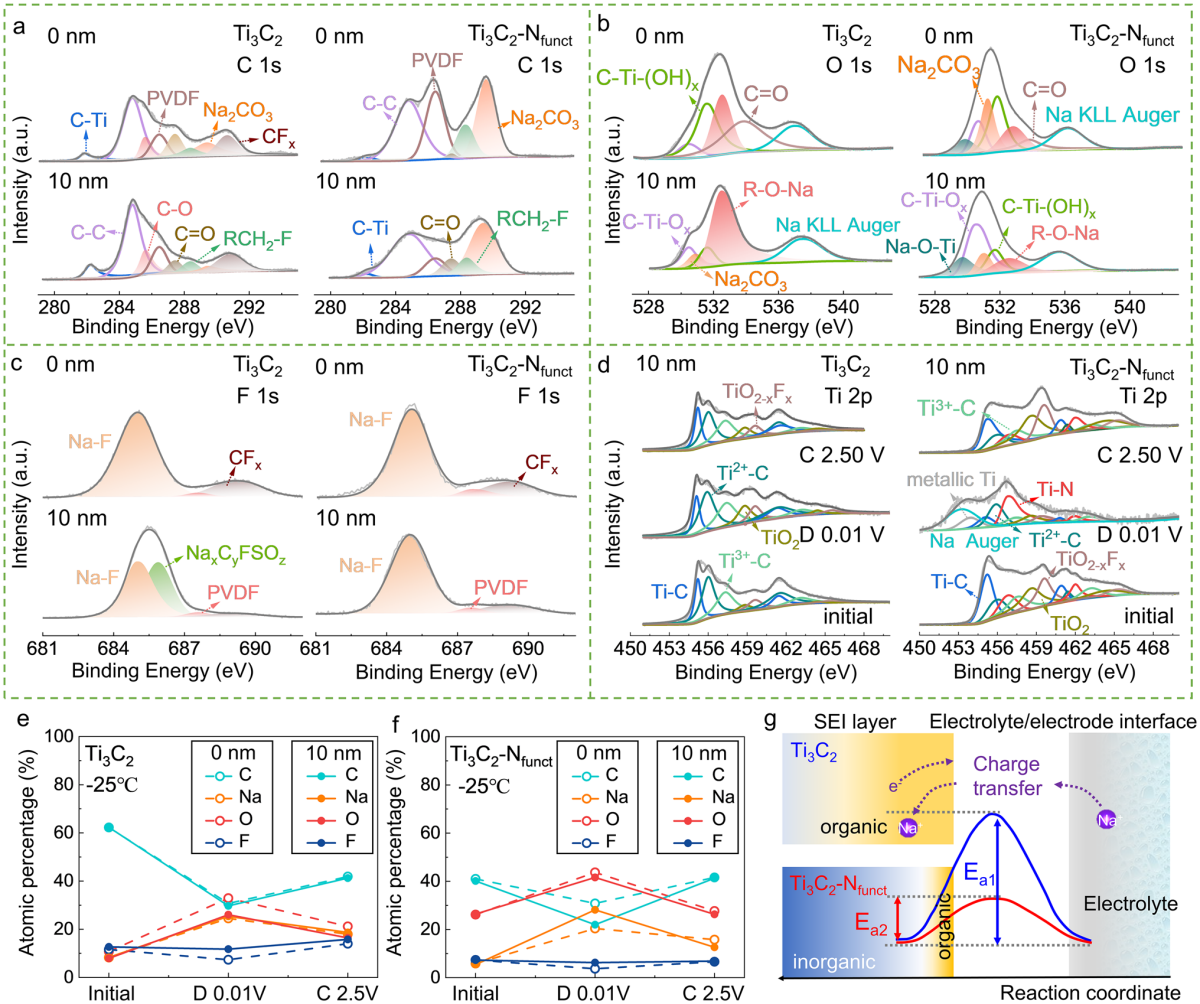
The surface composition analysis of Ti_3_C_2_ and Ti_3_C_2_-N_funct_ electrodes operating at − 25 °C by XPS etching. XPS **a** C 1*s*, **b** O 1*s* and **c** F 1*s* spectra of the Ti_3_C_2_ and Ti_3_C_2_-N_funct_ electrodes after first discharging to 0.01 V. **d** Ti 2*p* spectra for Ti_3_C_2_ and Ti_3_C_2_-N_funct_ at different states of charge (SOC) after Ar^+^ etching of 10 nm. The atomic percentage of C, Na, O, F of **e** Ti_3_C_2_ and **f** Ti_3_C_2_-N_funct_ electrodes at different SOC. **g** Illustration of the SEI compositions formed on Ti_3_C_2_ and Ti_3_C_2_-N_funct_ electrodes and a comparison of the charge transfer energy barriers

SEI is a crucial composition on electrode surface to affect the ion transport, which determines the electrochemical performance at low-T. To get deep insight of the SEI composition, high-resolution XPS of C 1*s*, O 1*s*, F 1*s*, and Na 1*s* spectra were collected from the electrode surface and in-depth of 10 nm with Ar^+^ sputtering for both Ti_3_C_2_ and Ti_3_C_2_-N_funct_ anodes (after discharging to 0.01 V at − 25 °C), as shown in Figs. 5a–c and S23. The C 1*s* spectra are fitted using peaks with binding energies of 284.2 (C–Ti), 284.8 (C–C), 285.6 (C–O), 286.3 (CH_2_–CF_2_), 287.4 (C=O), 288.3 (RCH_2_–F), 289.5 (O–C=O), and 290.8 (–CF_2_–) eV, which are consistent with Ti_3_C_2_, the main reduction products of diglyme solvent, Na_2_CO_3_ and PVDF [57]. These are also corresponding to the peaks located at 530.6 (C–Ti–O_x_), 531.0 (Na_2_CO_3_), 531.7 (C–Ti–(OH)_x_), 532.6 (R–O–Na), and 533.8 (C=O) eV (Fig. 5b). Due to the reduction of TiO_2_, the peak at 529.6 eV (Na–O–Ti) belonging to Na_x_TiO_2_ could be observed in O 1*s* spectra of Ti_3_C_2_-N_funct_ [58]. The peak located at 685.0 eV in F 1*s* spectra representing Na-F bond and the Na 1*s* spectra shows a peak at 1072.2 eV (Na–F and Na–O), demonstrating the existence of sodium compounds (Figs. 5c and S23) [51, 59]. Note that, the peak at 686.0 eV in F 1*s* spectra is belonging to the complex fluorosulfate, which is decomposed from NaCF_3_SO_3_ salt. Such a phenomenon could also be observed in the S 2*p* spectra (Figs. 5d and S24). Therefore, it can be inferred that the SEI layers formed on Ti_3_C_2_ and Ti_3_C_2_-N_funct_ anodes are composed of both organic compounds (RCH_2_ONa and ROCO_2_Na) and inorganic compounds (NaF and Na_2_CO_3_). The complex fluorosulfate is distributed near the surface of Ti_3_C_2_ electrode. It is noteworthy that there is a difference between SEI formed on the two electrodes, where the organic compounds are the main components in the SEI of Ti_3_C_2_ electrode and the inorganic compounds are dominated in the SEI of Ti_3_C_2_-N_funct_ electrode.

The compositions of SEI layer vary during the charge–discharge process. Figures 5e, f and S25 demonstrate the elemental compositions of SEI at different states of charge (SOC) and depths of Ti_3_C_2_ and Ti_3_C_2_-N_funct_ anodes. For both electrodes, the C and F concentrations decrease during the discharge process and increase when charging back to 2.5 V, while the trends of the O and Na concentrations are opposite. It could be ascribed to the formation and reconstruction of SEI layer during the discharge and charge process. Moreover, there is a difference between Ti_3_C_2_ and Ti_3_C_2_-N_funct_ electrodes when discharging to 0.01 V. For the Ti_3_C_2_-N_funct_ electrode, the C concentration decreases sharply with sputtering depths increasing, while the Na concentration increases and the O concentration decreases slightly. This phenomenon indicates that there is a thin organic compounds layer distributed near the surface and the inorganic compounds dominate the interior of the SEI. In contrast, there is no obvious change of the C and Na concentrations in the SEI of the Ti_3_C_2_ electrode. This means the organic compounds are distributed homogeneously in the SEI on the Ti_3_C_2_ electrode. Beneficial from the low charge transfer resistance of inorganic compounds as reported [58], the Ti_3_C_2_-N_funct_ electrode delivers lower activation energy during the interfacial charge transfer process as illustrated in Fig. 5g, which is consistent with the above dynamic analysis.

Moreover, the Ti 2*p* spectra of Ti_3_C_2_ and Ti_3_C_2_-N_funct_ at different SOC after Ar^+^ etching to a depth of 10 nm are shown in Fig. 5d. The Ti 2*p* spectra of Ti_3_C_2_-N_funct_ have greatly changed after discharged to 0.01 V, wherein the ratio of Ti^2+^-C increases and the peaks of Ti^2+^-C, Ti^3+^-C, and Ti-N shift to the lower binding energy, indicating the reduced valence of Ti and the formation of Ti–O–Na and Na–N–Ti interaction [60]. After the extraction of Na^+^, the partial Ti^2+^ is oxidized to Ti^3+^ when charged back to 2.5 V, demonstrating the reversible redox reactions of the Ti^3+^/Ti^2+^ couple. However, as for the Ti_3_C_2_ electrode, the ratio of Ti^2+^-C and Ti^3+^-C in Ti 2*p* spectra nearly maintained during the discharging and charging process. Such a phenomenon demonstrates that tailoring N-terminals on Ti_3_C_2_ provides more active sites for the redox reaction and induces more charge transfer during the charge/discharge process, leading to higher capacity. Overall, due to the inorganic component of SEI layer and more active sites for redox reaction, it is easy to understand the reason why Ti_3_C_2_-N_funct_ delivers better sodium storage performance than that of Ti_3_C_2_ as shown in Fig. 3.

### Na^+^ Storage Mechanism of Ti_3_C_2_-N_funct_ at Low Temperature

**Fig. 6:**
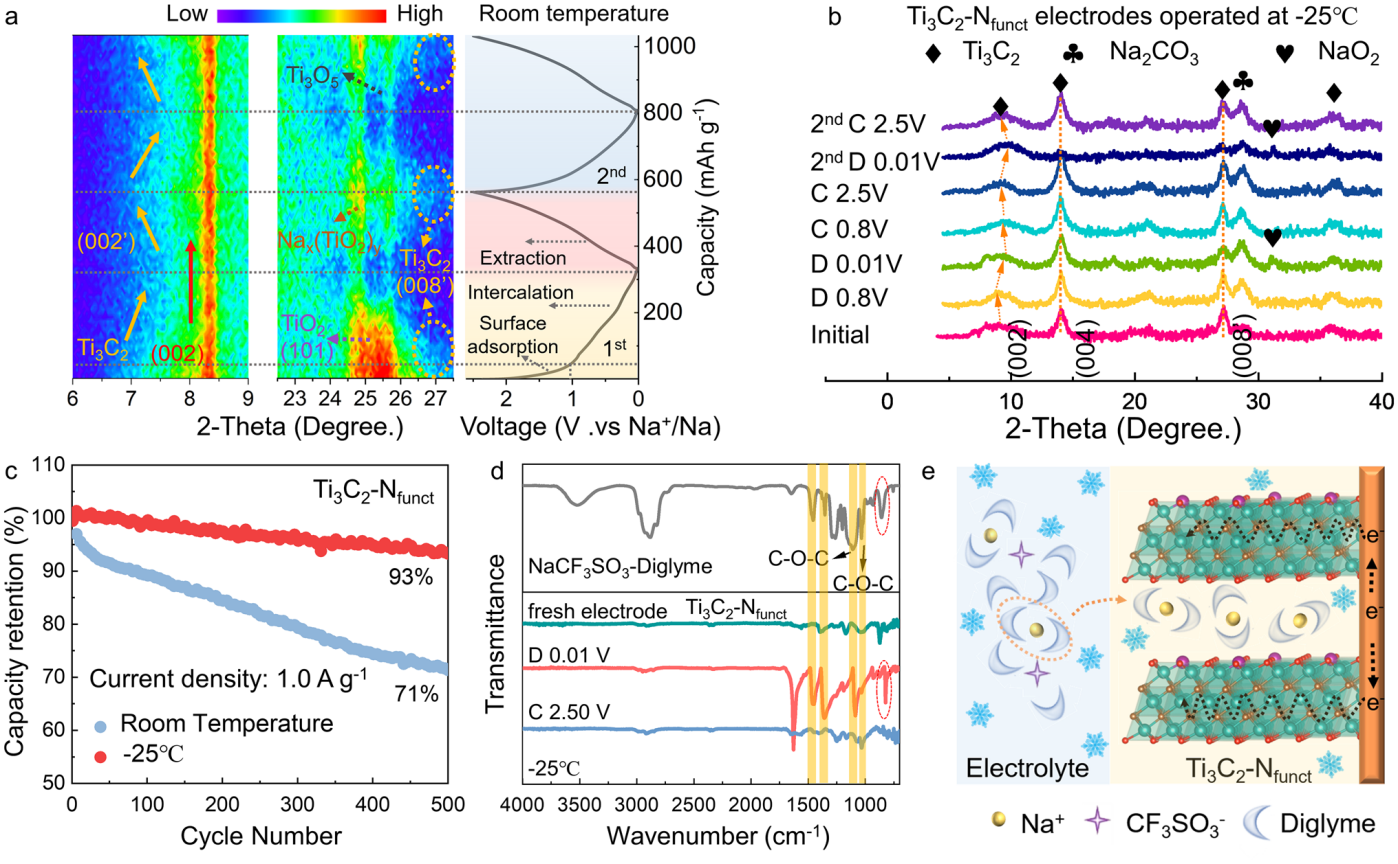
**a** In situ XRD patterns of Ti_3_C_2_-N_funct_ operating at room temperature with 2θ ranging from 6.0° to 9.0° and 22.5°–27.5°, the first two charge/discharge curves corresponding to the in situ XRD patterns. **b** Ex situ XRD patterns of Ti_3_C_2_-N_funct_ electrodes operating at − 25 °C. **c** Comparison of cycling performance for Ti_3_C_2_-N_funct_ operating at room temperature and − 25 °C. **d** Ex situ FTIR spectra of Ti_3_C_2_-N_funct_ electrodes. **e** Illustration of Na^+^-solvent co-intercalation behavior in Ti_3_C_2_-N_funct_ at low-T

How do Ti_3_C_2_-N_funct_ participate in the electrochemical reactions at low-T? Cycling performance is used to compare the Na^+^ storage capability of Ti_3_C_2_-N_funct_ at different conditions (Fig. 6c). Interestingly, Ti_3_C_2_-N_funct_ electrode delivers stable cycling performance at − 25 °C than that at room temperature. To facilitate a deep understanding of the sodiation behavior of Ti_3_C_2_-N_funct_, in situ and ex situ XRD measurements were made to demonstrate the structure reversibility during the charging–discharging process under different conditions. In situ XRD patterns with 2θ ranging from 6.0° to 9.0° and 22.5°–27.5° of Ti_3_C_2_-N_funct_ operating at room temperature are displayed in Fig. 6a. According to the discharge curve, the sodiation process can be divided into surficial adsorption and intercalation. Although the (002) peak belonging to Ti_3_C_2_ fixes at 8.3° during the continuous discharging–charging process, the (002′) peak shifts to a higher angle during discharging, and then could not return when get the fully charged state. Moreover, the (008’) peak disappears during discharging and reemerges at the charged state, which could be attributed to the intercalation of Na^+^. These intercalated Na^+^ will attract the MXene layers to each other, leading to a reduced interlayer space [61, 62]. The obvious variation for the layer structure of Ti_3_C_2_-N_funct_ during the charging–discharging process would lead to unstable cycling performance at room temperature. The reflections located between 22.5° and 27.5° are attributed to the sodiation process of TiO_2_, in which TiO_2_ is transformed to sodium titanates (JCPDS No. 78-1590), titanium suboxide (JCPDS No. 72-2101) [58]. However, the TiO_2_ particles forming during the tailoring process didn’t contribute to the increased capacity of Ti_3_C_2_-N_funct_ as shown in Fig. S26. In addition, TiO_2_-N_mix_ (deriving from the annealing product of TiO_2_ mixed with CTAB powders) exhibits an unpleasant performance than pure TiO_2_, further proving that the improved performance of Ti_3_C_2_-N_funct_ exactly stemmed from tailoring surficial N-terminals rather than the contribution of TiO_2_ (Fig. S27).

To explore the Na^+^ storage mechanism at low-T, ex situ XRD patterns of Ti_3_C_2_-N_funct_ electrodes operating at − 25 °C were recorded (Fig. 6b). Interestingly, after aging for 1 h in Na-ion half-cell at − 25 °C, the potential of Ti_3_C_2_-N_funct_ reduced 0.05 V (Fig. S28a). It could be attributed to the chemical pre-sodiation of Ti_3_C_2_-N_funct_ during the aging process, leading to the broaden of (002) peak, the shift of (004) peak of Ti_3_C_2_-N_funct_, and the disappearance of peaks of anatase TiO_2_ (Fig. S28b). It has been reported that the anatase TiO_2_ would transform into amorphous structure when sodiated at low temperature [63]. The widened (002) peak shifts to a lower angle when firstly discharged to 0.8 V, and then it returns to the higher angle at the fully discharged state. Two new lattice peaks are detected at 28.5° and 31.4°, corresponding to Na_2_CO_3_ and NaO_2_. Different from operating at room temperature, the interlayer spacing of Ti_3_C_2_-N_funct_ is enlarged before discharging to 0.8 V, and then it shrinks during subsequent discharging process at low-T. Such phenomenon could be correlated to the intercalation of the solvent molecules [61, 64]. Moreover, the slight the variation of (002) peak indicates the stable structure of Ti_3_C_2_-N_funct_, leading to outstanding cycling performance at low-T. The elemental mapping of Ti_3_C_2_-N_funct_ at a deep-discharged state is exhibited in Fig. S29 further prove the Na^+^ intercalation at low-T.

It is believed that the desolvation process is the rate-determining step at low-T due to the high desolvation energy barrier [65, 66]. As previous studies have indicated that there is an ion–solvent co-intercalation behavior in graphitic structure when operated in the ether-based electrolytes with high solvation energy [67, 68], we speculate that the graphite-like MXenes possessing larger interlayer spacing might deliver the similar ion–solvent co-intercalation behavior to circumvent the desolvation process to achieve fast kinetics at low-T. To confirm the hypothesis, the ex situ FTIR spectra of Ti_3_C_2_-N_funct_ electrodes during sodiation and desodiation are collected. The FTIR spectra of the electrolyte (1 M NaCF_3_SO_3_ in diglyme) and pure solvent (diglyme) are compared in Fig. S30, demonstrating that the peak at 858 cm^−1^ belonging to the solvated Na^+^ [69]. The characteristic peaks of the solvated Na^+^ appear at the full discharged state and disappear when charged back to 2.5 V in the ex situ FTIR spectra of Ti_3_C_2_-N_funct_ and Ti_3_C_2_ (Figs. 6d and S31). Our previous work shows that if the solvent is simply adsorbed on the surface of the material, its signal is independent of the state of charge and could be detected during the whole charging–discharging process [70]. In Ti_3_C_2_-N_funct_, the characteristic peaks of diglyme solvent are strongly associated with SOC, suggesting that Na^+^ and solvent might possess similar migration behavior. Therefore, integrating these evidences into account, we deduced that Ti_3_C_2_-N_funct_ possesses Na^+^-solvent co-intercalation behavior during the charge transfer process as illustrated in Fig. 6e, which could avoid the high desolvation energy barrier to realize the fast-charging ability at low-T.

## Conclusions

In summary, we propose and demonstrate that tailoring nitrogen terminals on Ti_3_C_2_ through the interlayer confined strategy is crucial to enable high-performance SIBs at low temperature. N atoms derived from the decomposition of confined CTAB molecules directly substitute the surface terminals, which tailor the in-plane structure of Ti_3_C_2_. The interfacial kinetics and energy storage mechanism at − 25 °C of Ti_3_C_2_-N_funct_ are investigated. It is found that tailoring nitrogen terminals could boost Na^+^ diffusion kinetics and lower charge transfer barrier by the synergistic effects of large interlayer spacing, charge redistribution, and strong adsorption, empowering Ti_3_C_2_-N_funct_ with higher Na^+^ diffusion coefficient and a 10% reduction in activation energy at low-T. The inorganic compounds in the SEI on Ti_3_C_2_-N_funct_ are beneficial for Na^+^ transfer. Moreover, the ion–solvent co-intercalation behavior endows Ti_3_C_2_-N_funct_ with fast-charging ability at low-T. As expected, the Ti_3_C_2_-N_funct_ anodes deliver high capacity retention, fast-charging ability (charging 80% capacity within 18 min), and ultra-long lifespan (5000 cycles with a capacity retention of 80.9%) at − 25 °C, far exceeding that of pristine Ti_3_C_2_. The assembled Ti_3_C_2_-N_funct_//NVPF full cells also deliver high energy density and cycling stability at − 25 °C. This work opens avenues for the development of other 2D materials in constructing high-energy storage systems at low temperatures.

## Supplementary Information

Below is the link to the electronic supplementary material.

Supplementary file 1 (PDF 2934 KB)
