# Supplementary material for: Tailoring Nitrogen Terminals on MXene Enables Fast Charging and Stable Cycling Na-Ion Batteries at Low Temperature
Source: Nanomicro Lett. 2022 Jul 9;14:143. doi: 10.1007/s40820-022-00885-7 (PMC9271150; doi:10.1007/s40820-022-00885-7)
Supplement: Supplementary file 1 — (PDF 2934 KB) [file 40820_2022_885_MOESM1_ESM.pdf]

Supporting Information for

Tailoring Nitrogen Terminals on MXene Enables Fast Charging and Stable Cycling Na-Ion Batteries at Low-Temperature

Yang Xia[1], Lanfang Que[2,*], Fuda Yu[2], Liang Deng[1], Zhenjin Liang[3], Yunshan Jiang[1], Meiyan Sun[1], Lei Zhao[1,*], and Zhenbo Wang[1,4,*]

[1]MIIT Key Laboratory of Critical Materials Technology for New Energy Conversion and Storage, State Key Lab of Urban Water Resources and Environment, School of Chemistry and Chemical Engineering, Harbin Institute of Technology, Harbin 150001, P. R. China

[2]Engineering Research Center of Environment-Friendly Functional Materials, Ministry of Education, Institute of Materials Physical Chemistry, Huaqiao University, Xiamen 361021, P. R. China

[3]The Institute for Advanced Studies, Wuhan University, Wuhan 430072, P. R. China

[4]College of Materials Science and Engineering, Shenzhen University, Shenzhen 518071, P. R. China

*Corresponding authors. E-mail: quelanfang@126.com (Lanfang Que); leizhao@hit.edu.cn (Lei Zhao); wangzhb@hit.edu.cn (Zhenbo Wang)

S1 Materials

All chemicals were of analytical grade and used directly without any purification. Ti_3AlC_2 MAX powders were purchased from Jilin 11 technology Co., Ltd. Lithium fluoride (LiF), hexadecyltrimethylammonium bromide (CTAB), titanium dioxide (TiO_2) were purchased from Shanghai Aladdin reagent Co. Ltd. Hydrochloric acid (HCl) was purchased from Tianjin Kemiou Chemical Reagent Co., Ltd. Electrolyte were purchased from Suzhou Dodochem Ltd.

S1.1 Synthesis of Ti_3C_2 MXenes

Ti_3C_2 was synthesized by liquid etching process. Firstly, 1.6 g LiF powders were added into 40 mL 9.0 M HCl solution. To totally dissolve the salt, the mixture was heated up to 45 °C and stirred for 5 min. Secondly, 1 g Ti_3AlC_2 powders were added into the mixture and kept at 45 °C for 72 h until Al was completely etched. Thirdly, the black suspension was washed with deionized water until the pH of supernatant reached 6, then the sediment was collected. Afterward, the sediment was dispersed in 100 mL of deionized water under sonication with argon bubbling for 60 min and ice bathing, then the dark green Ti_3C_2 solution (4 mg mL^{-1}) was collected. Finally, Ti_3C_2 powders were obtained by freeze-drying the dark green solution.

S1.2 Synthesis of Ti_3C_2-CTconfined and Ti_3C_2-Nfunct MXenes

0.1 g CTAB powders were added into 50 mL Ti_3C_2 solution. The mixture was sonicated for 1 h and then stirred for 24 h at 40 °C. Next, the mixture was freeze-dried to gather the solid product labeled as Ti_3C_2-CTconfined. Finally, Ti_3C_2-Nads was calcinated at 350 °C for 3 h under flowing Ar atmosphere to obtain Ti_3C_2-Nfunct samples.

S1.3 Synthesis of Ti_3C_2-Nmix and TiO_2-Nmix

0.1 g CTAB powders were mixed with 0.2 g Ti_3C_2 powders and nano TiO_2 powders, respectively. These mixtures were groud for 10 min. These mixed powders were calcinated at 350 °C under flowing Ar atmosphere to obtain Ti_3C_2-N_{mix} and TiO_2-N_{mix} samples, repectively.

S2 Physical Characterization

The structure evolution was analyzed by in-situ high-temperature XRD and in-situ electrochemical XRD. The crystal structure was obtained by using Bruker D8 Advanced diffractometer with a Cu $K_{\alpha 1}$ radiation source (λ=1.5406 Å). The structure evolution in the Ti_3C_2-N_{ads} sample was also recorded by a thermal gravity analyzer (TGA, NETZSCH TG 209F3) at the heating rate of 5 °C min^{-1} in Ar atmosphere. The morphologies and microstructures were observed by a field emission SEM (S-4800) and a field emission TEM (JEM-2100), respectively. XPS analysis was conducted by using an Al K_{α} radiation (Thermo Scientific K-Alpha+). The FTIR spectra was recorded by Scientific Nicolet iS5 (THERMO) with a resolution of 0.8 cm^{-1}.

S3 Electrochemical Measurements

To evaluate the Na^+ storage performance of these MXenes, coin-type (CR2025) half- cells were fabricated by coupling MXenes-based electrodes with metal sodium. In addition, the Ti_3C_2-N_{funct}//NVPF SIBs were assembled by combining the Ti_3C_2-N_{funct} anodes with NVPF cathodes. The MXenes-based electrodes and NVPF cathodes were both prepared by mixing active materials, super P and polyvinylidene fluoride solution (NMP as a solvent, mass percentage of 6.5%) with a mass ratio of 7:2:1. The slurry was coated on copper/aluminum foils and dried at 120 °C for 12 h. The electrode was punched into circle with diameters of 14 mm. The average mass loading of anode and cathode were 0.8-1.2 mg cm^{-2} and 1.8-2.0 mg cm^{-2}, respectively. For the full cells, the mass ratio of Ti_3C_2-N_{funct} and NVPF in SIBs is 2:1. The assembling process of half- cells and Ti_3C_2-N_{funct}//NVPF SIBs was carried in a glovebox filled with pure Ar. The Whatman glass fiber was selected as the separator. 1.0 M $NaCF_3SO_3$ dissolved in diglyme was used as an electrolyte at -25 °C. Galvanostatic charge-discharge tests were conducted at different current densities on NEWWARE battery testing system (Shenzhen, China). Cyclic voltammetry (CV) and electrochemical impedance spectroscopy (EIS) tests were measured by the electrochemical work station (CHI660E, Chenhua, Shanghai).

S4 Computational Details

Theoretical calculations were performed by using the CASTEP module of Materials Studio software package within the density functional theory (DFT) framework. The Perdew-Burke-Ernzerhof (PBE) functional under generalized gradient approximation (GGA-PBE) was adopted to describe the exchange correlation energy. With the application of the projector augmented wave (PAW) technique, the plane-wave energy cutoff was set to 598.7 eV. Moreover, the Brillouin zone was represented by Monkhorst−Pack special k-point mesh of 2×3×1 for geometry optimizations. To calculate the Na^+ adsorption energy, a 3×2×1 supercell of $Ti_3C_2O_2$ was constructed. In order to avoid the interactions between the adjacent layers, a vacuum layer of 20 Å in the Z direction was created. When performing the structure optimizations, the system is regarded as converged when the force per atom is less than 0.01 eV/Å. The adsorption energies (E_{ad}) for Na atom on functional Ti_3C_2 monolayer surfaces are defined as:

$$E_{ad} = \frac{E_{MXene-Na} - E_{MXene} - nE_{Na}}{n} \quad \text{(S1)}$$

in which E_{MXene} is the energy of the 3×2×1 supercell of the functionalized MXene monolayer, $E_{MXene\text{-}Na}$ stands for the total energy of sodium atoms adsorbed on the MXene monolayer, and E_{Na} refers to the energy of per sodium atom in a bulk bcc crystal. Furthermore, the climbing-

image nudged elastic band (CI-NEB) method was used to determine the diffusion energy barrier and the minimum energy pathways for Na^+ diffusion in double layers of $Ti_3C_2O_2$ and $Ti_3C_2O_{1.83}N_{0.17}$.

S5 Supplementary Figures and Tables

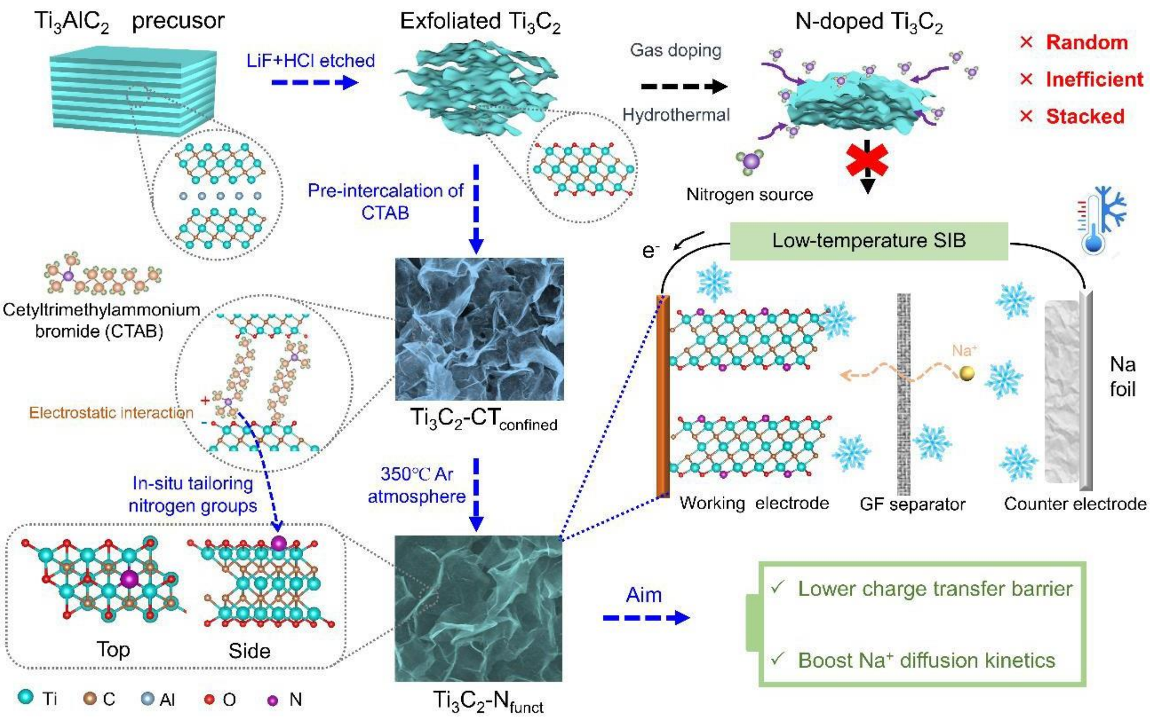

Fig. S1 Illustration of traditional nitrogen element doping methods and interlayer confined strategy for tailoring nitrogen groups on Ti_3C_2 to achieve high-performance SIBs at low temperature

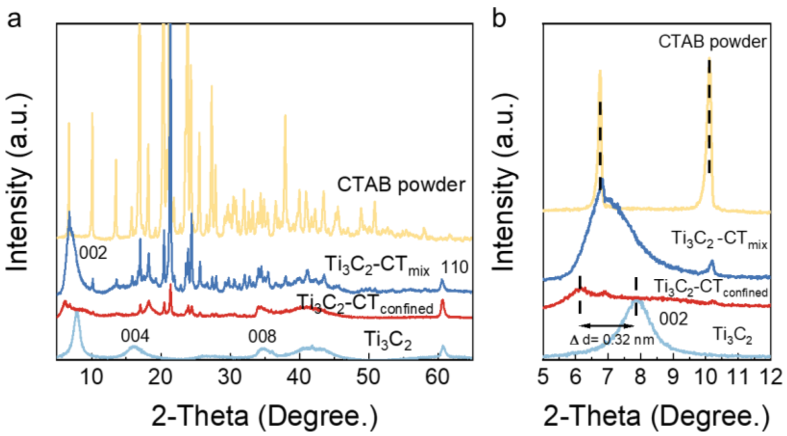

Fig. S2 (**a**)The XRD patterns of CTAB powders, Ti_3C_2, Ti_3C_2-$CT_{confined}$ and Ti_3C_2-CT_{mix}. (**b**) Detailed views of the XRD pattern in (**a**) with 2θ ranging from 5-12°

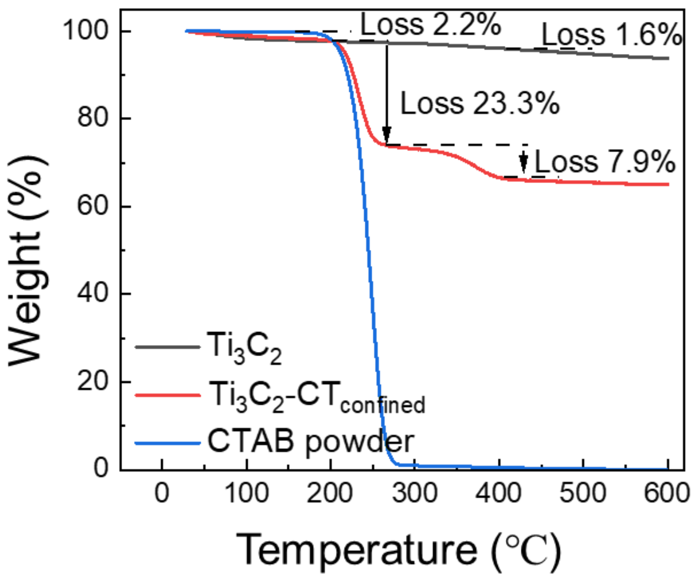

Fig. S3 TGA curves of Ti_3C_2, CTAB powder and Ti_3C_2-$CT_{confined}$ from 25-600 °C

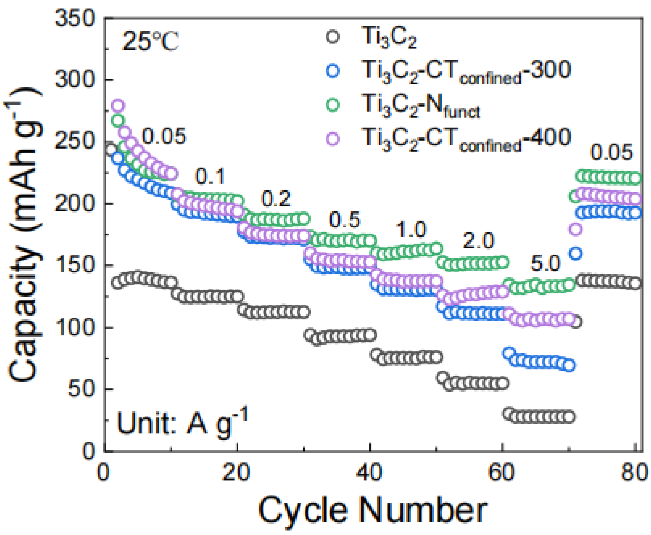

Fig. S4 Rate performance of Ti_3C_2, Ti_3C_2-$CT_{confined}$-300 (Ti_3C_2-$CT_{confined}$ annealing under 300 °C), Ti_3C_2-N_{funct}, and Ti_3C_2-$CT_{confined}$-400 (Ti_3C_2-$CT_{confined}$ annealing under 400 °C)

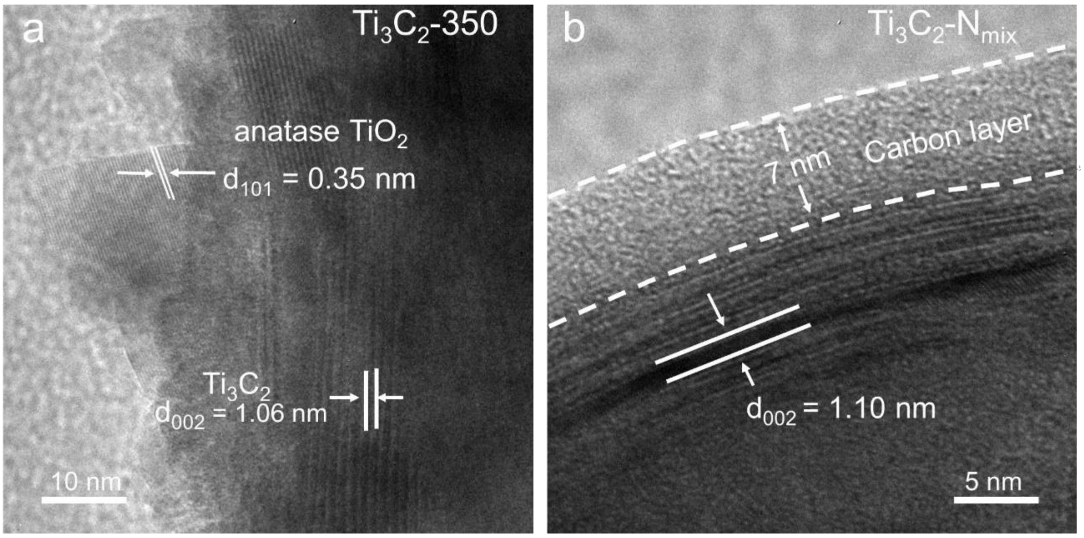

Fig. S5 HRTEM images of (**a**) Ti_3C_2-350, (**b**) Ti_3C_2-N_{mix}

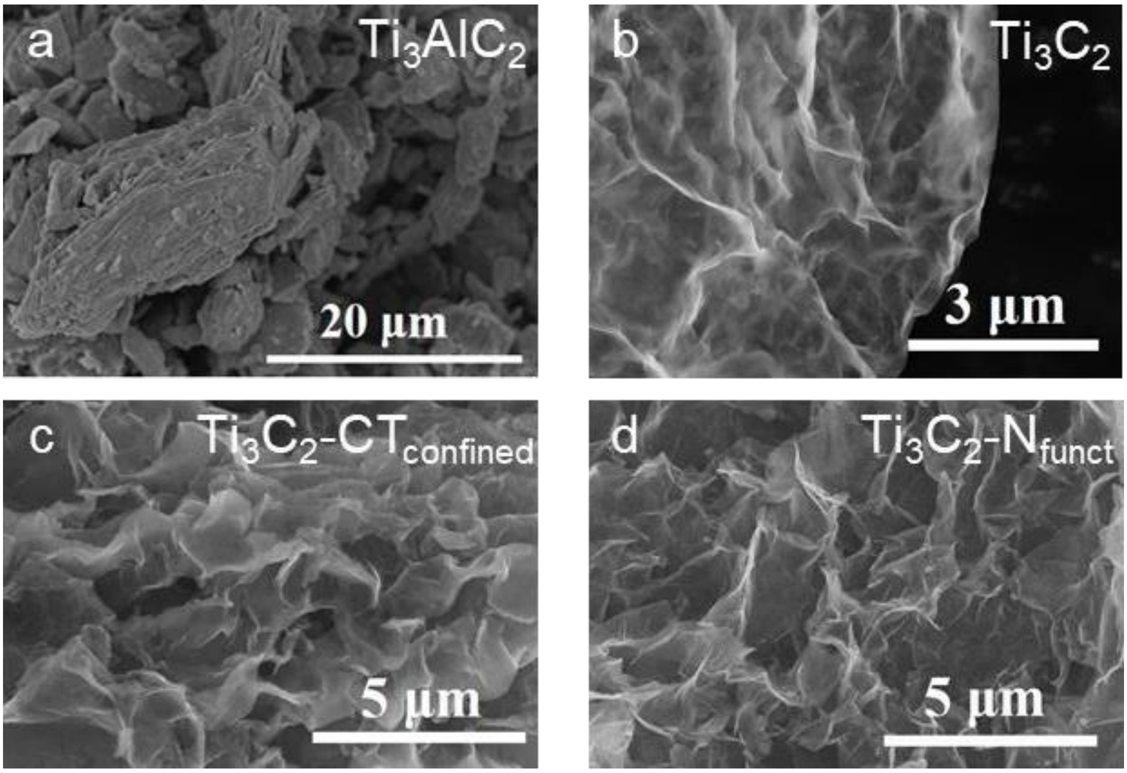

Fig. S6 SEM images of (**a**) Ti_3AlC_2, (**b**) Ti_3C_2, (**c**) Ti_3C_2-CTconfined and (**d**) Ti_3C_2- N_{funct}

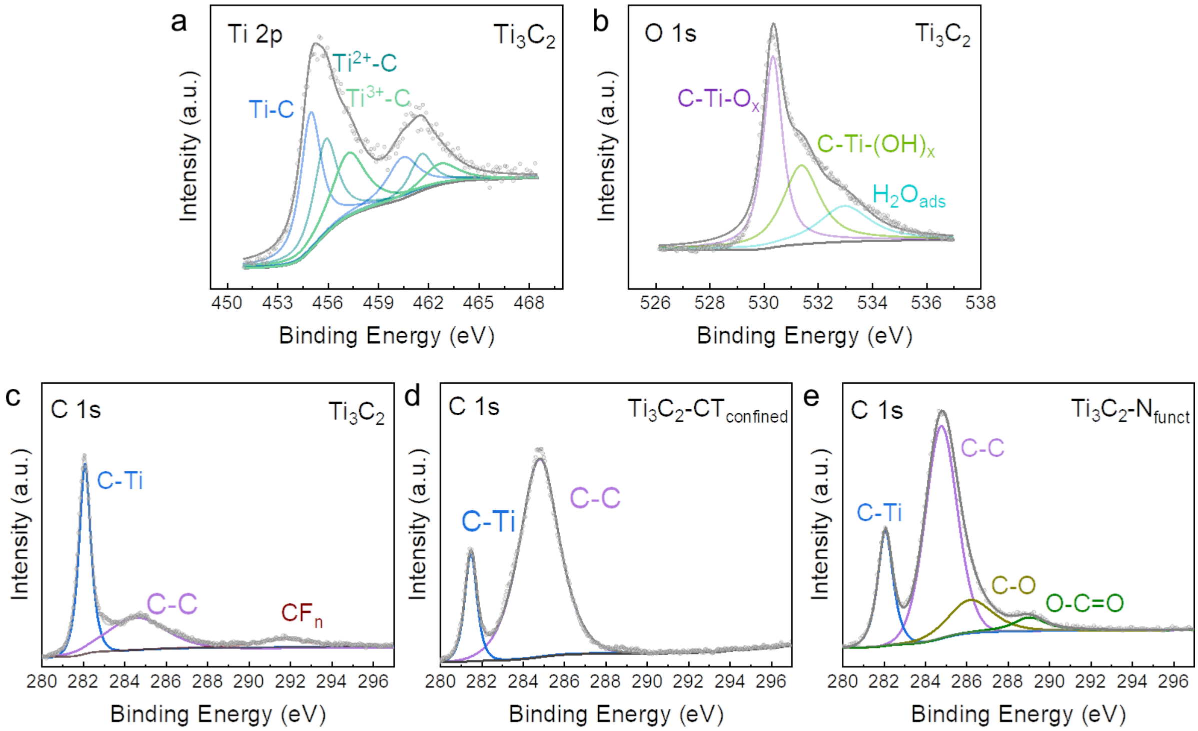

Fig. S7 High resolution XPS spectra of (**a**) Ti 2p, (**b**) O 1s and (**c**) C 1s for Ti_3C_2 sample. C 1s spectra of (**d**) Ti_3C_2-$CT_{confined}$ and Ti_3C_2-N_{funct}

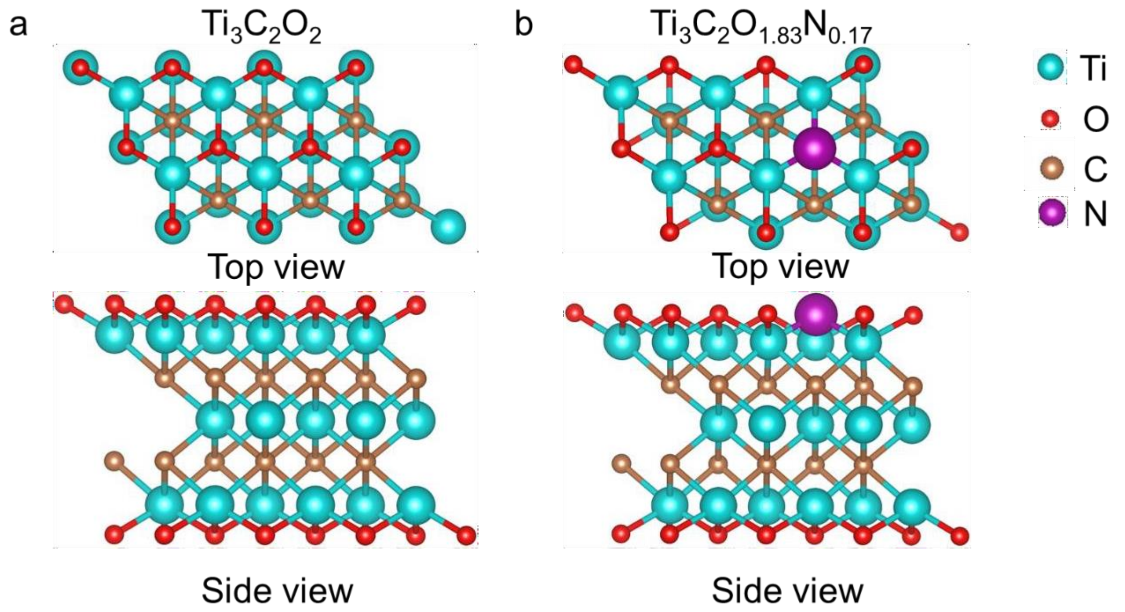

Fig. S8 Optimized geometries of Ti_3C_2 monolayer with different surficial components of top and side view: (**a**) $Ti_3C_2O_2$ and (**b**) $Ti_3C_2O_{1.83}N_{0.17}$

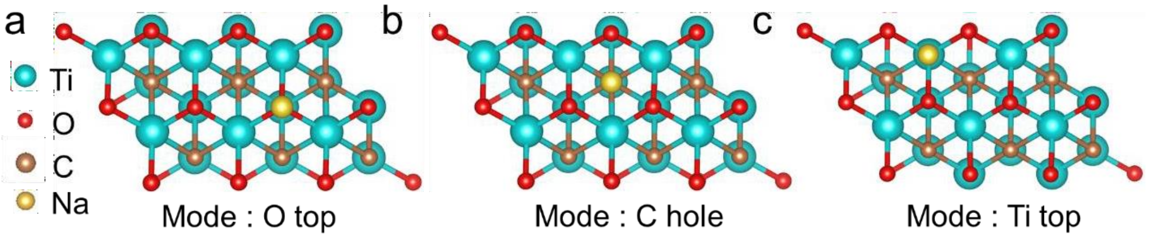

Fig. S9 Optimized geometries structures for a Na atom adsorption on the different sites on $Ti_3C_2O_2$ monolayer: (**a**) top of O atom, (**b**) top of C atom, (**c**) top of Ti atom

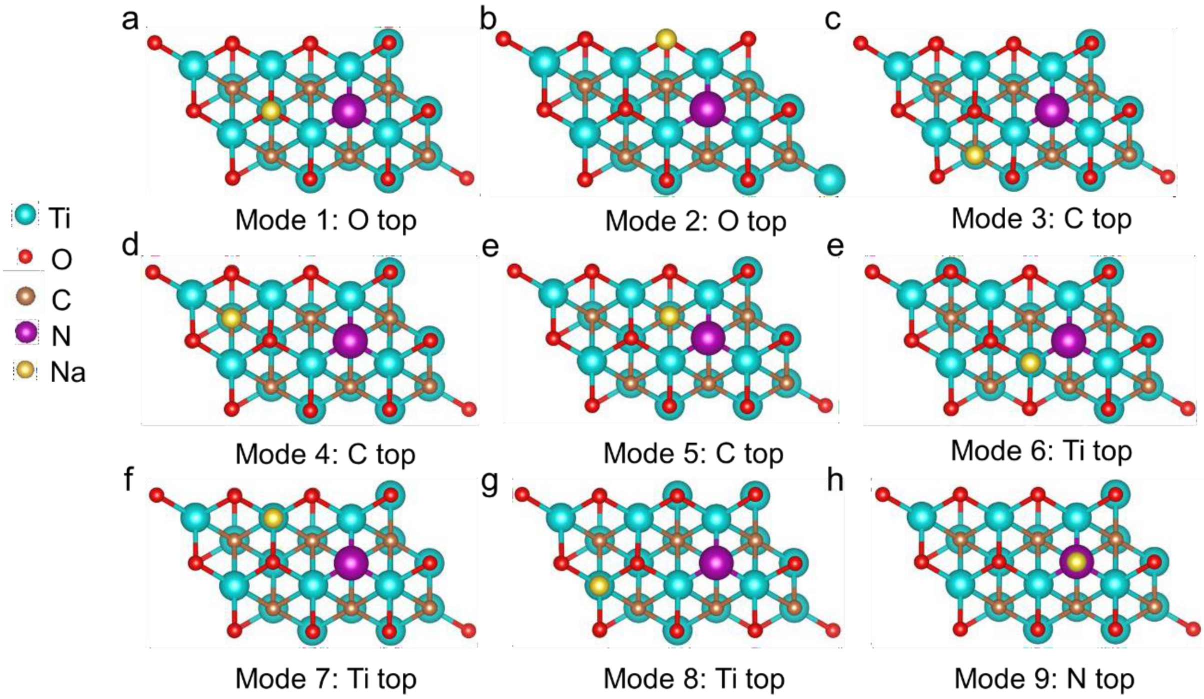

Fig. S10 Optimized geometries structures for a Na atom adsorption on the different sites on $Ti_3C_2O_{1.83}N_{0.17}$ monolayer: (**a-b**) top of O atom, (**c-e**) top of C atom, (**e-g**) top of Ti atom, (**h**) top of N atom

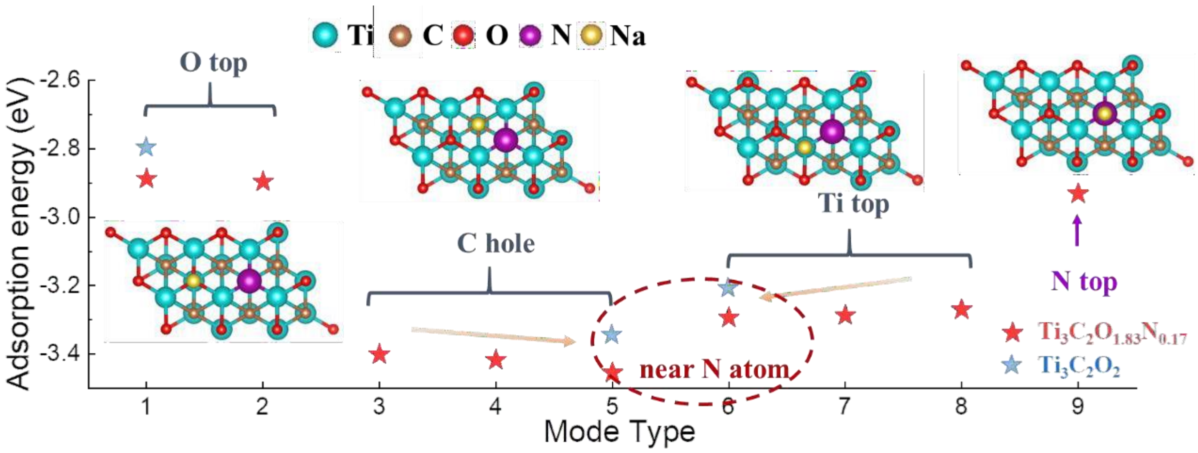

Fig. S11 Comparison of the adsorption energy of a Na atom on $Ti_3C_2O_2$ and $Ti_3C_2O_{1.83}N_{0.17}$

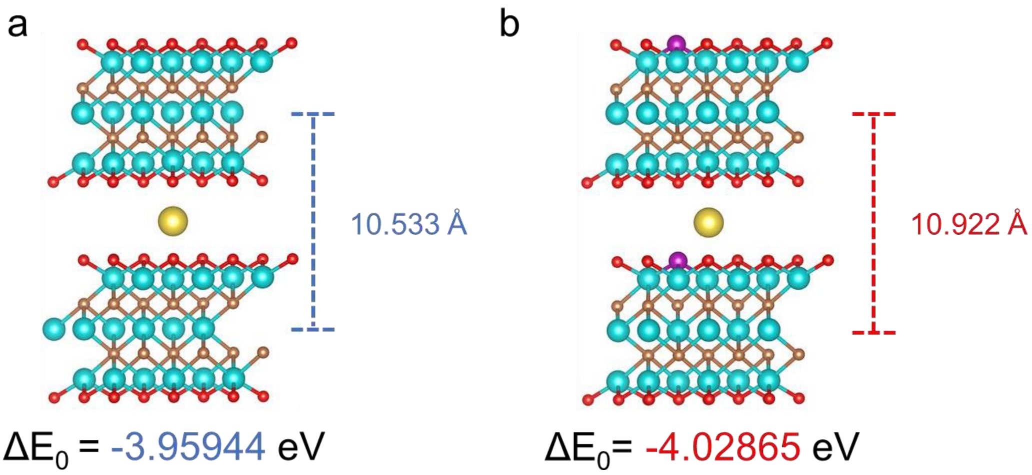

Fig. S12 Formation energy of a Na atom in double layers of (**a**) $Ti_3C_2O_2$ and (**b**) $Ti_3C_2O_{1.83}N_{0.17}$

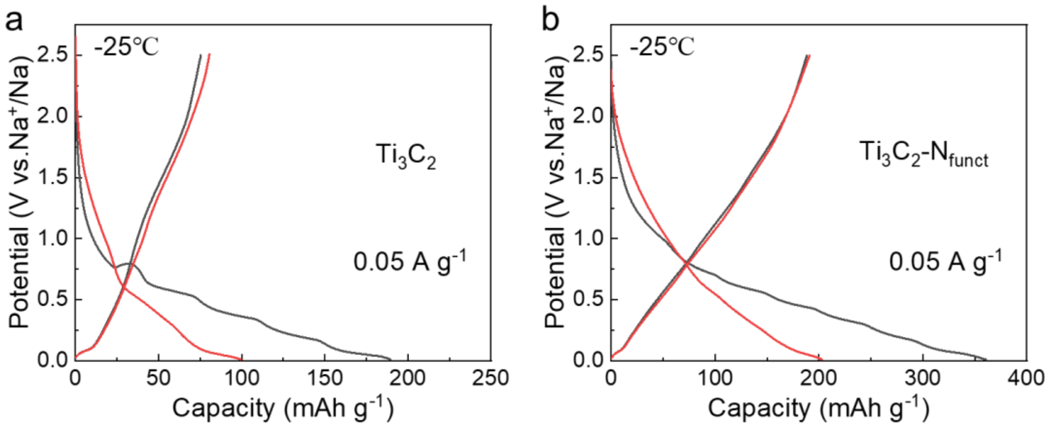

Fig. S13 First two cycles charge/discharge curves of (**a**) Ti_3C_2 and (**b**) Ti_3C_2-N_{funct} at 0.05 A g^{-1} under low-T

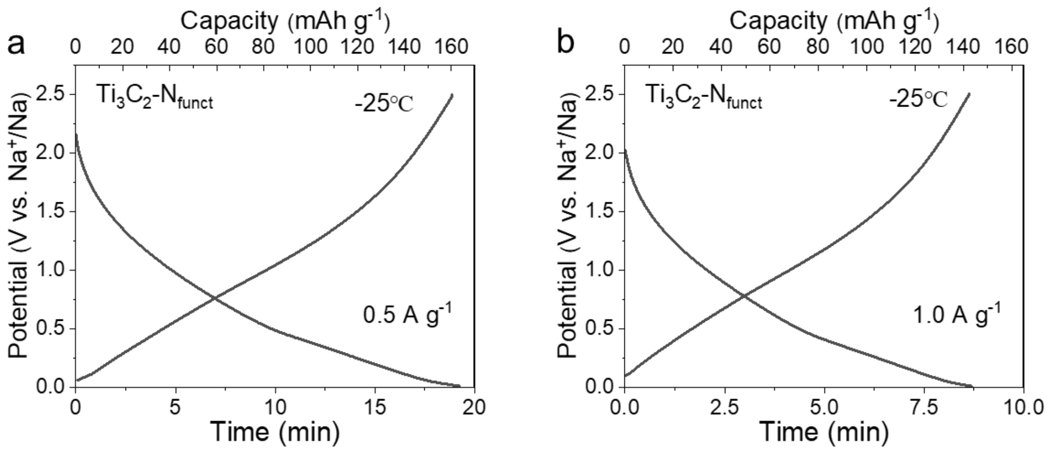

Fig. S14 Charge/discharge curves of Ti_3C_2-N_{funct} at (**a**) 0.5 and (**b**) 1.0 A g^{-1} under -25℃

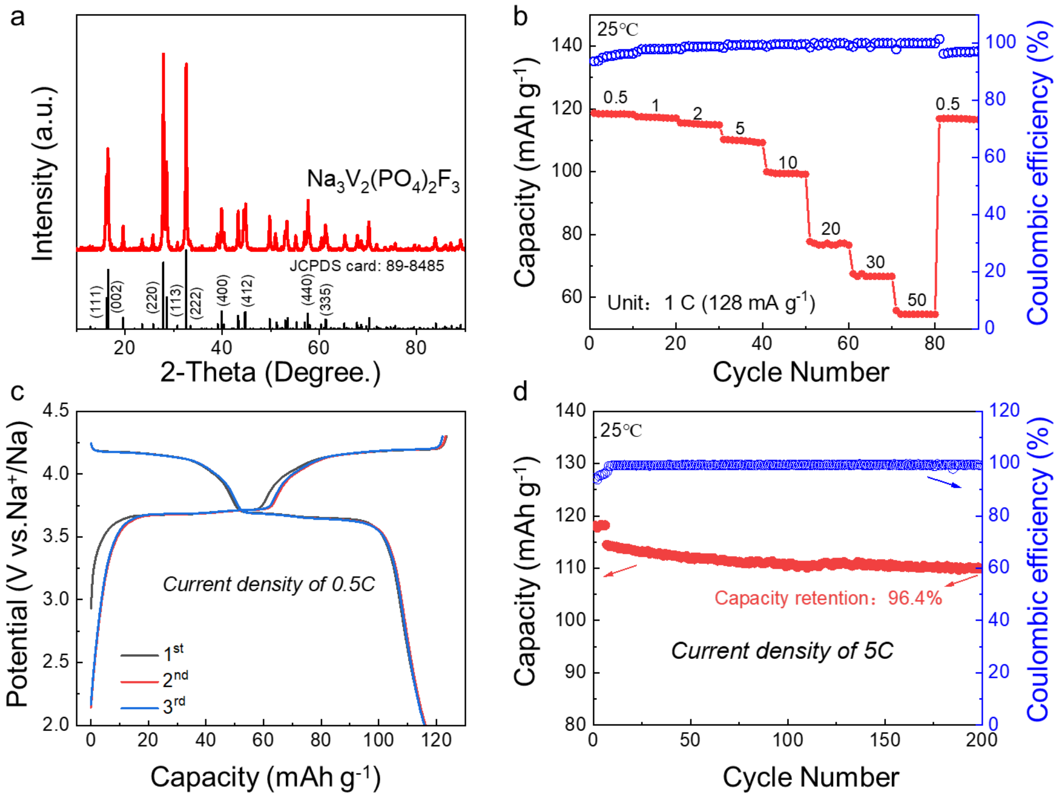

Fig. S15 (**a**) XRD pattern of NVPF cathode. The electrochemical performance of NVPF cathode at room temperature. (**b**) Rate performance. (**c**) The first three charge/discharge curves of NVPF. (**d**) The cycling performance

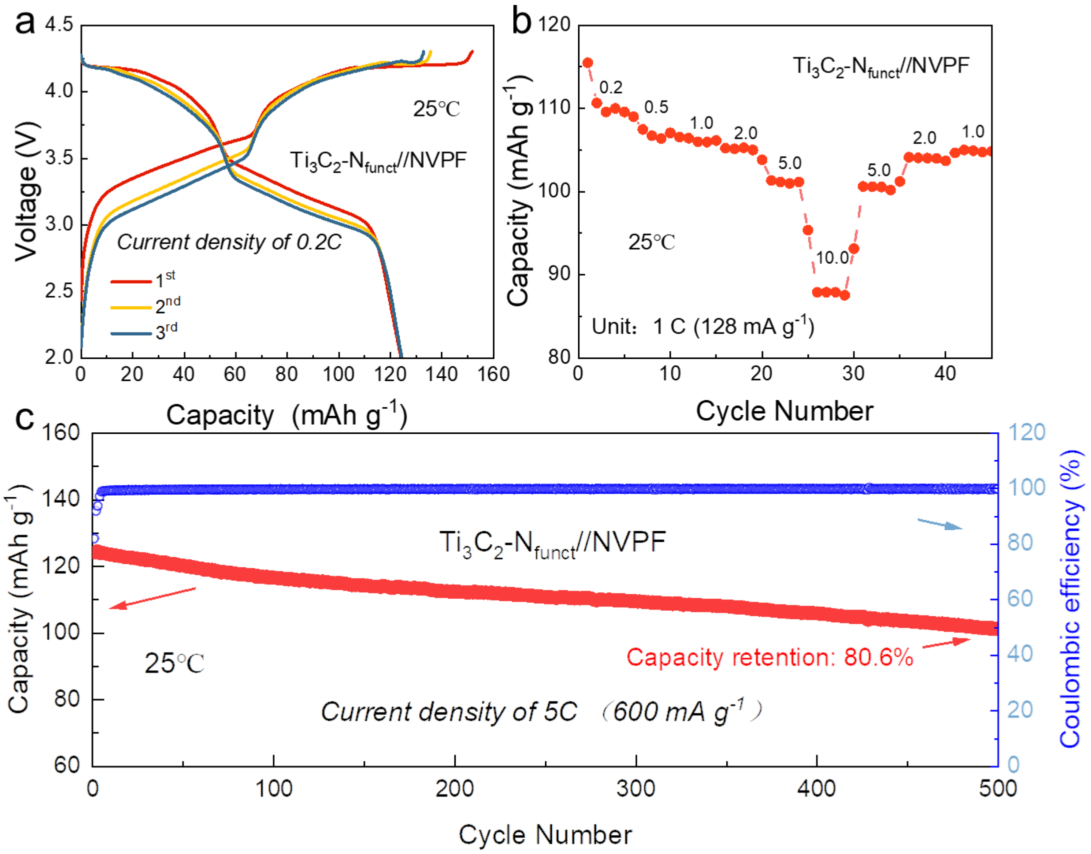

Fig. S16 Electrochemical performance of the Ti_3C_2-N_{funct}//NVPF full cell at room temperature. (**a**) The first three charge/discharge curves of Ti_3C_2-N_{funct}//NVPF full cell at 0.2C (1C represents 0.128 mA g^{-1}). (**b**) Rate performance and (**c**) cycling performance

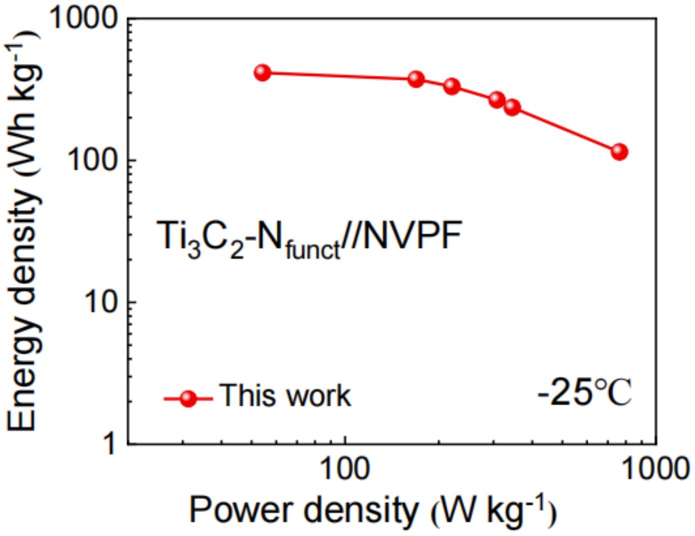

Fig. S17 The Ragone plot of Ti_3C_2-Nfunct//NVPF full cell operating at -25 °C

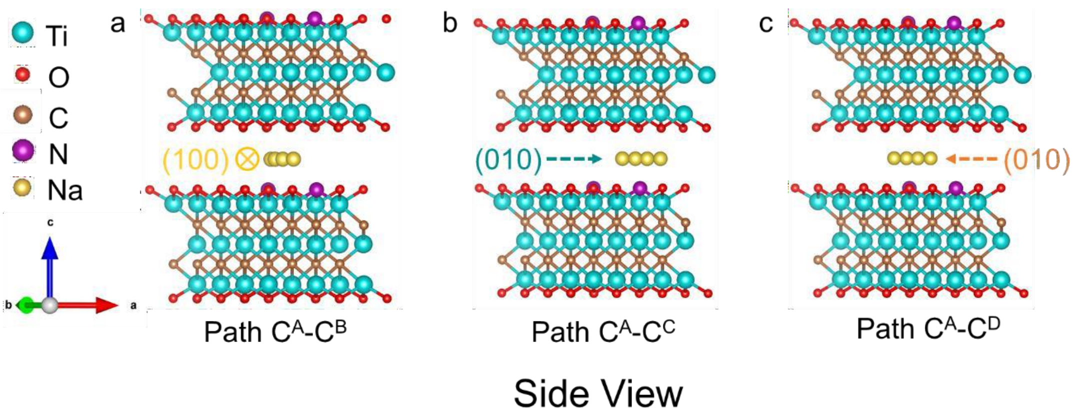

Fig. S18 Side view of three diffusion directions of Na atom between two layers of $Ti_3C_2O_{1.83}N_{0.17}$

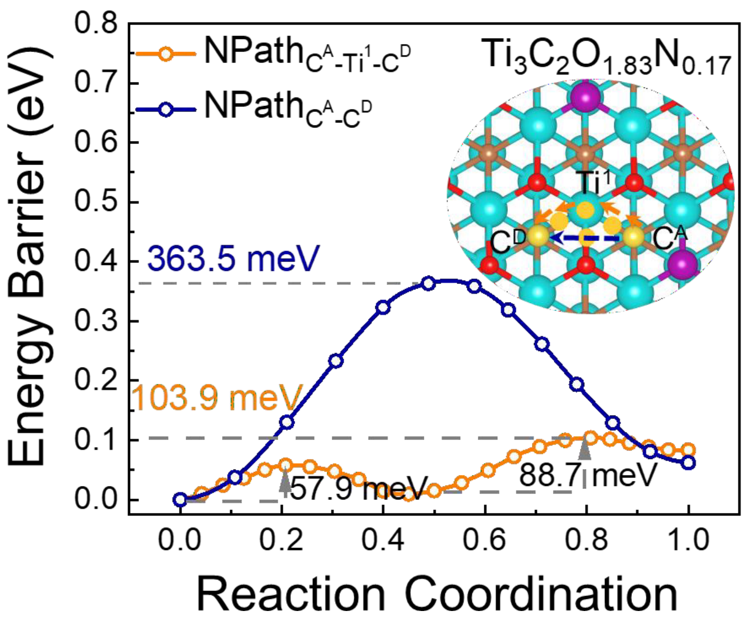

Fig. S19 Compared the energy barrier of Na^+ diffusion paths from C^A site to C^D site in double layers of $Ti_3C_2O_{1.83}N_{0.17}$

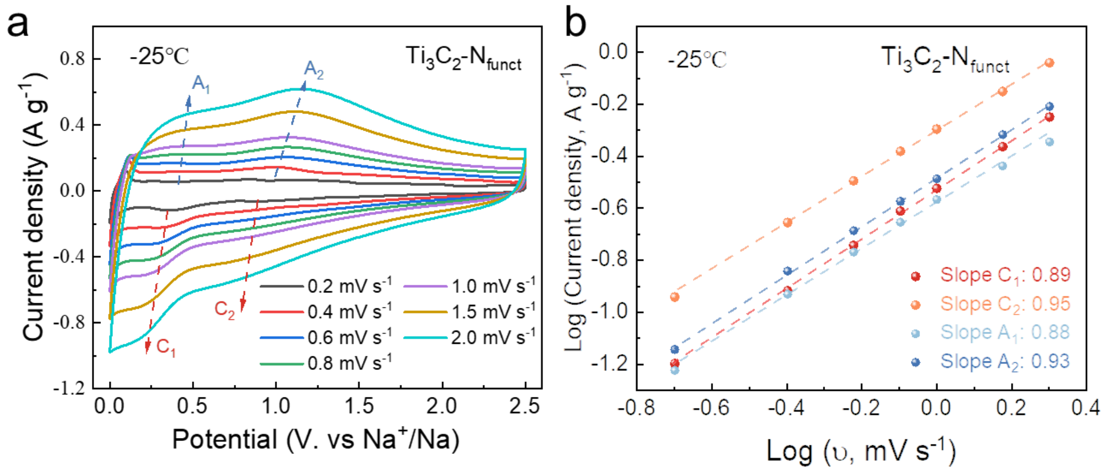

Fig. S20 (**a**) CV curves of Ti_3C_2-N_{funct} at different scan rates from 0.2 to 2.0 mV s^{-1} at -25 °C. (**b**) Log(i) vs Log(v) plots of the cathodic and anodic current response at four peaks shown in (**a**)

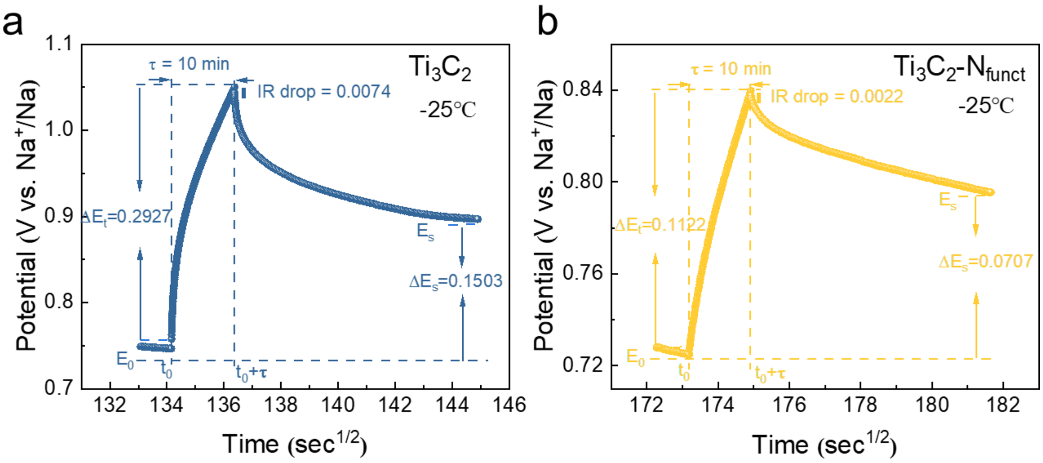

Fig. S21 $t^{1/2}$ vs. V plots of the (**a**) Ti_3C_2 and (**b**) Ti_3C_2-N_{funct}

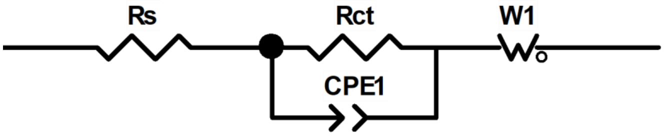

Fig. S22 Equivalent circuit model of Ti_3C_2 and Ti_3C_2-N_{funct} electrodes

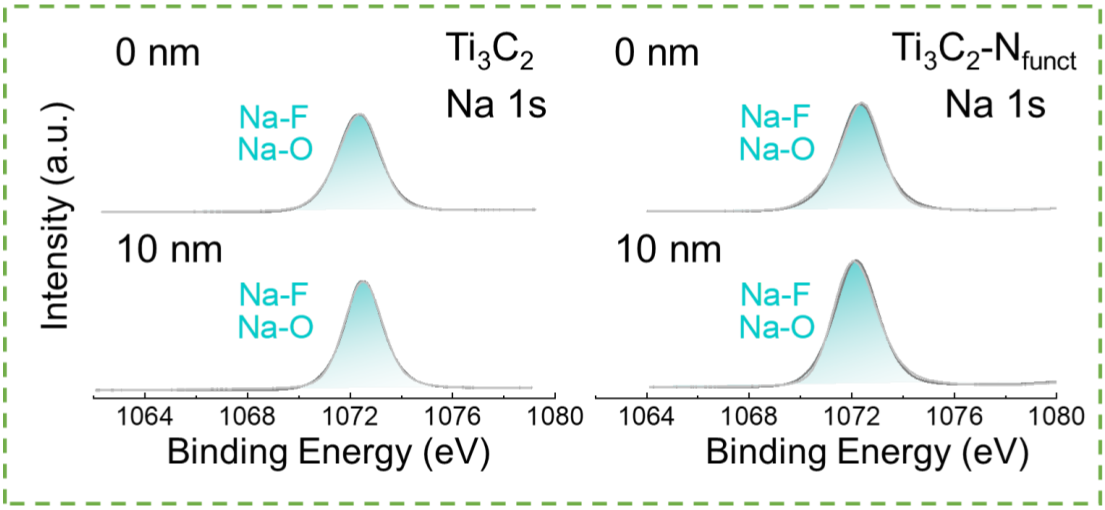

Fig. S23 XPS Na 1s spectra of the surface and in-depth of 10 nm with Ar^+ sputtering for both Ti_3C_2 and Ti_3C_2-N_{funct} electrodes after discharging to 0.01 V

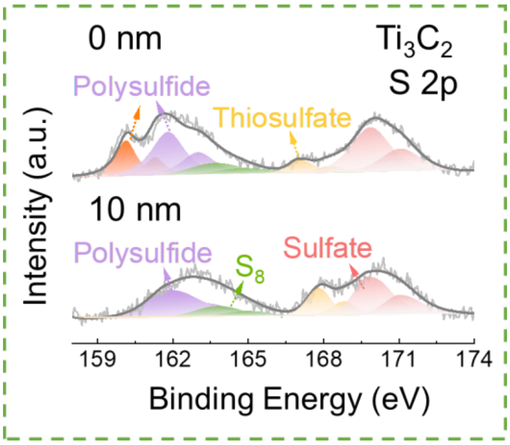

Fig. S24 XPS S 2p spectra of the surface and in-depth of 10 nm with Ar^+ sputtering for Ti_3C_2 electrodes after discharging to 0.01 V

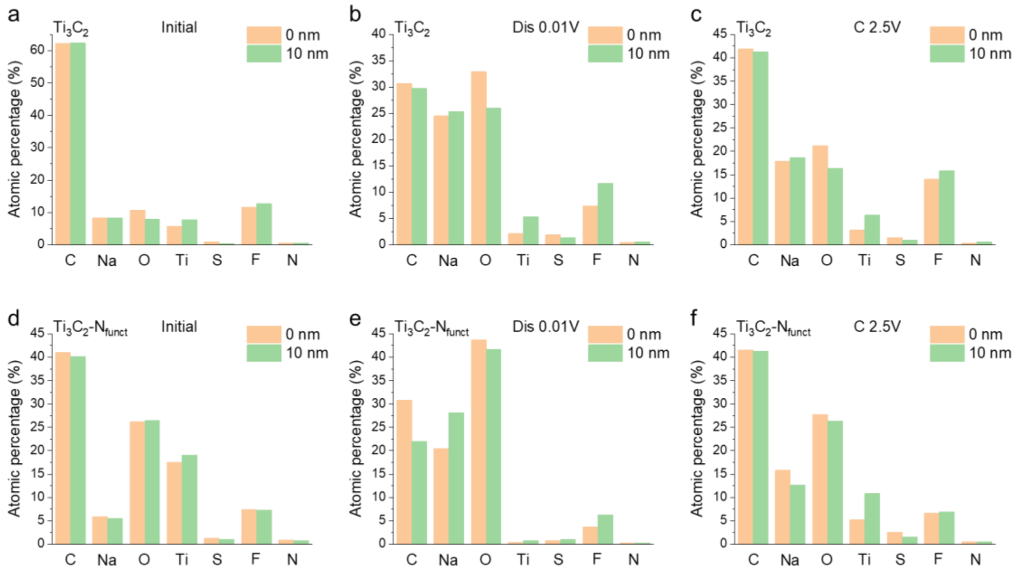

Fig. S25 Atomic fractions of Na, C, O, F, Ti, N and S elements in (**a-c**) Ti_3C_2 and (**d-f**) Ti_3C_2-N_{funct} electrodes at different SOC

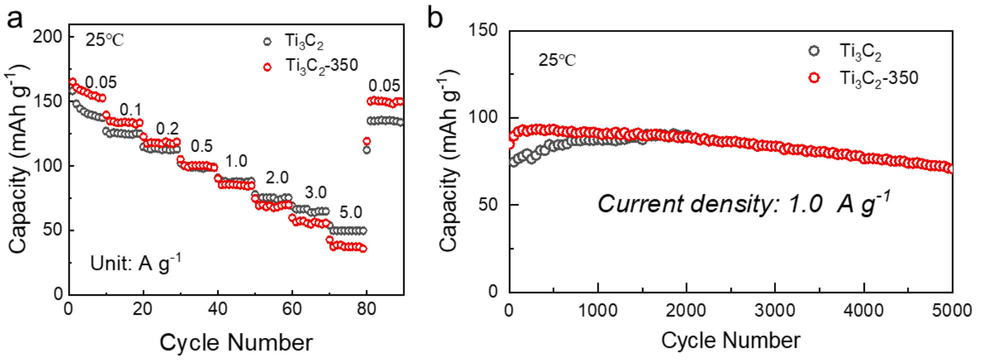

Fig. S26 Electrochemical performance of Ti_3C_2 and Ti_3C_2-350 at 25°C: (**a**) rate performance; (**b**) cycle stability at 1.0 A g^{-1}

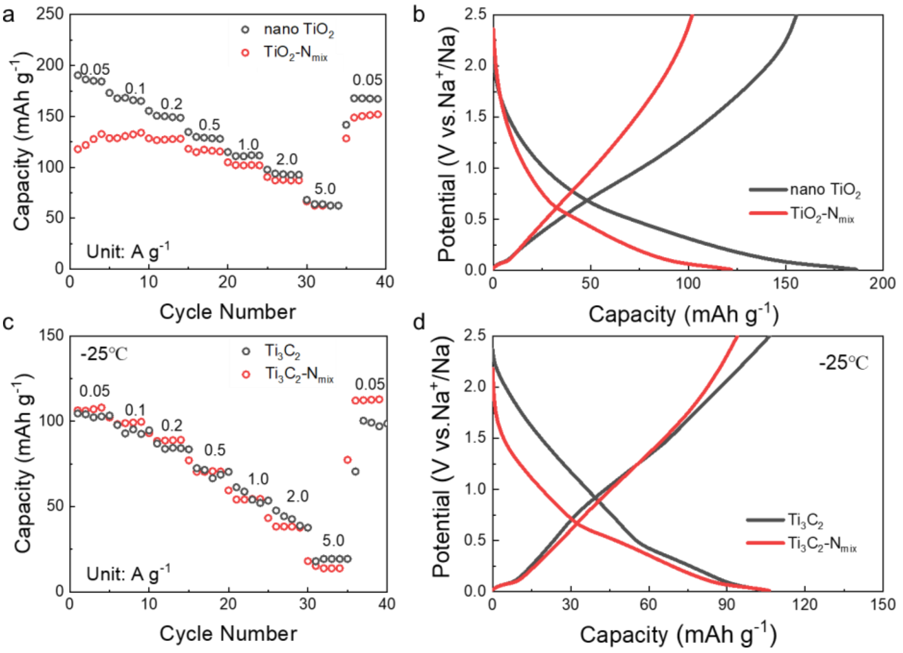

Fig. S27 Electrochemical performance. (**a**) Rate ability of nano-TiO_2 and TiO_2-N_{mix}. (**b**) Charge/discharge curves of nano-TiO_2 and TiO_2-N_{mix} at 0.05 A g^{-1}. (**c**) Rate ability of Ti_3C_2 and Ti_3C_2-N_{mix}. (**d**) Charge/discharge curves of Ti_3C_2 and Ti_3C_2-N_{mix} at 0.05 A g^{-1}

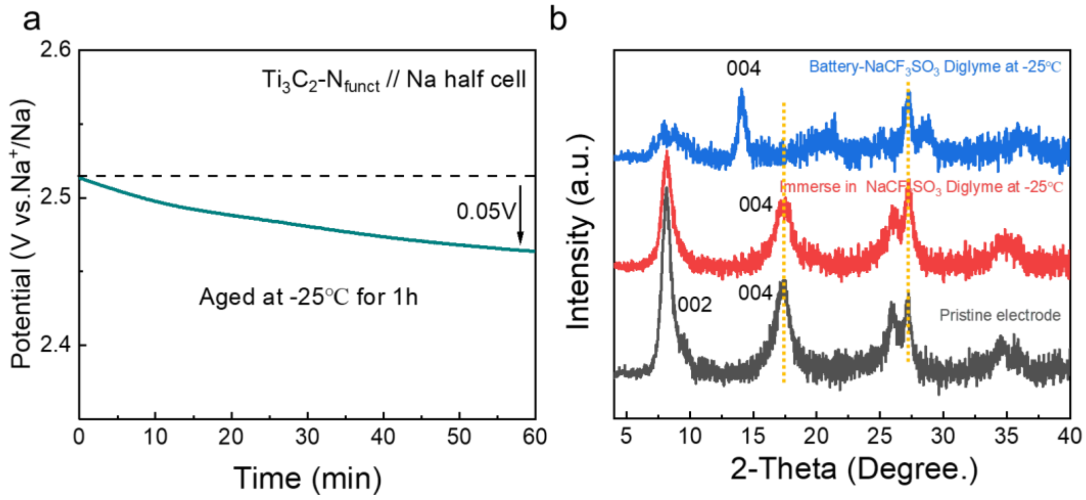

Fig. S28 (**a**) The time-voltage curve of Ti_3C_2-N_{funct} // Na half-cell aging at -25°C for 1h. (**b**) XRD patterns of Ti_3C_2-N_{funct} electrodes: pristine, immersing in electrolyte and assembling in half cell

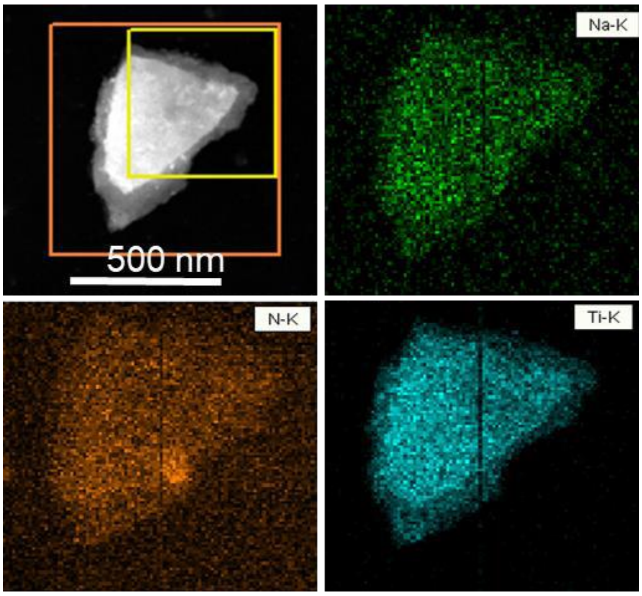

Fig. S29 STEM image and corresponding Na, N and Ti elements mappings of fully discharged Ti_3C_2-N_{funct}

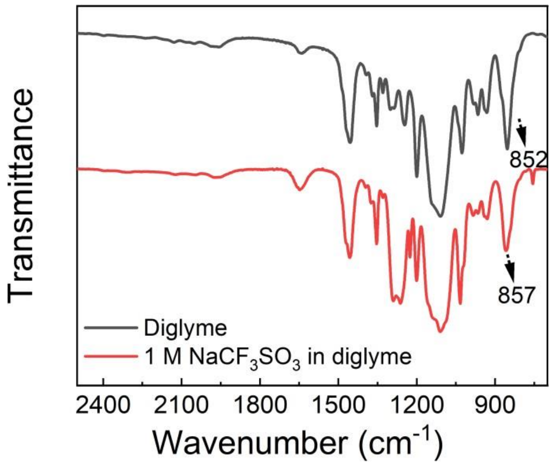

Fig. S30 FTIR spectra of diglyme and 1 M $NaCF_3SO_3$ in diglyme

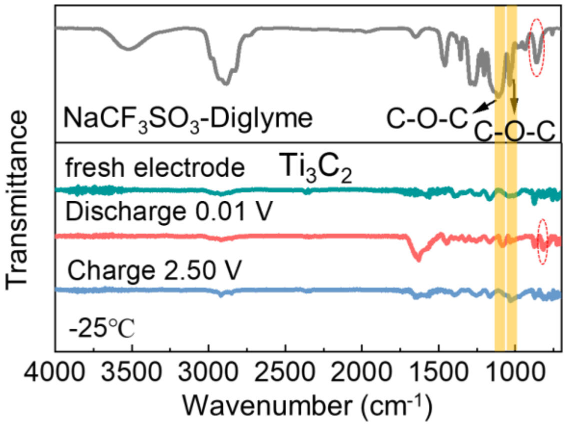

Fig. S31 *Ex-situ* FTIR spectra of Ti_3C_2 anodes

Table S1 Atomic ratio of elements in Ti_3C_2-$CT_{confined}$ and Ti_3C_2-N_{funct} samples calculated by XPS

Sample	Element	Atomic ratio (%)
Ti_3C_2-$CT_{confined}$	Ti	25.71
	C	52.10
	O	16.76
	N	3.63
	Br	1.79
Ti_3C_2-N_{funct}	Ti	24.69
	C	22.33
	O	51.87
	N	1.05
	Br	0.07

Table S2 Comparison of stability with other materials of LIBs and SIBs at low temperature

Sample		Temperature (℃)	Current density (A g^{-1})	Capacity retention (%)
3D Se/graphene composite[S1]	SIBs	-25	2.0	90.4(1000 cycles)
FeS_2@G@CNF[S2]	SIBs	-20	0.2	66.7(100 cycles)
NTP/C-F[S3]	SIBs	-20	2.6	84.2(1000 cycles)
LS-Sb@G[S4]	SIBs	-20	0.12	63.7(50 cycles)
S-LT[S5]	SIBs	-20	1.0	91(1200 cycles)
LTO/HG-3[S6]	LIBs	-25	1.75	89.4(500 cycles)
MoS_2/C[S7]	LIBs	-25	1.75	95.6(50 cycles)
GeO_X@MXene[S8]	LIBs	-40	0.24	83.4(100 cycles)
$Ti_3C_2T_X$(O)[S9]	LIBs	-20	0.1	85.8(1000 cycles)
**This work**	**SIBs**	**-25**	**1.0**	**80.9(5000 cycles)**
**Ti_3C_2-Nfunct//NVPF**	**Full cell**	**-25**	**0.06**	**88.9(200 cycles)**

Table S3 The R_{ct} fitted values of Ti_3C_2 and Ti_3C_2-N_{funct}

Sample	Temperature (°C)	R_{ct} (Ohm)
Ti_3C_2	25	2.273
	10	3.169
	0	3.811
	-10	7.592
	-25	8.060
Ti_3C_2-N_{funct}	25	2.834
	10	3.663
	0	3.914
	-10	7.720
	-25	8.953

Supplementary References

[S1] Y.Y. Wang, B.H. Hou, J.Z. Guo, Q.L. Ning, W.L. Pang et al., An ultralong lifespan and low-temperature workable sodium-ion full battery for stationary energy storage. Adv. Energy Mater. **8**(18), 1703252 (2018). https://doi.org/10.1002/aenm.201703252

[S2] C.M. Chen, Y.C. Yang, X. Tang, R.H. Qiu, S.Y. Wang et al., Graphene-encapsulated FeS_2 in carbon fibers as high reversible anodes for Na+ /K+ batteries in a wide temperature range. Small **15**(10), 1804740 (2019). https://doi.org/10.1002/smll.201804740

[S3] X.H. Rui, X.H. Zhang, S.T. Xu, H.T. Tan, Y. Jiang et al., A low-temperature sodium-ion full battery: superb kinetics and cycling stability. Adv. Funct. Mater. **31**(11), 2009458 (2020). https://doi.org/10.1002/adfm.202009458

[S4] K.C. Huang, J.Z. Guo, H.H. Li, H.H. Fan, D.H. Liu et al., Layer-stacked Sb@graphene micro/nanocomposite with decent Na-storage, full-cell and low-temperature performances. J. Alloys Compd. **731**, 881-888 (2018). https://doi.org/10.1016/j.jallcom.2017.10.110

[S5] L.F. Que, F.D. Yu, Y. Xia, L. Deng, K. Goh et al., Enhancing Na-ion storage at subzero temperature via interlayer confinement of Sn^{2+}. ACS Nano **14**(10), 13765-13774 (2020). https://doi.org/10.1021/acsnano.0c05925

[S6] J.H. Jeong, M.J. O, K.C. Roh, Correlation between lithium-ion accessibility to the electrolyte-active material interface and low-temperature electrochemical performance. J. Alloys Compd. **856**, 158233 (2021). https://doi.org/10.1016/j.jallcom.2020.158233

[S7] X.Z. Liu, Y.H. Wang, Y.J. Yang, W.Lv, G. Lian et al., A MoS_2/carbon hybrid anode for high-performance Li-ion batteries at low temperature. Nano Energy **70**, 104550 (2020). https://doi.org/10.1016/j.nanoen.2020.104550

[S8] M.W. Shang, X. Chen, B.X. Li, J.J. Niu, A fast charge/discharge and wide-temperature battery with a germanium oxide layer on a Ti_3C_2 MXene matrix as anode. ACS Nano **14**(3), 3678-3686 (2020). https://doi.org/10.1021/acsnano.0c00556

[S9] Y.H. Wang, C. Ma, W.Q. Ma, W. Fan, Y. Sun et al., Enhanced low-temperature Li-ion storage in MXene titanium carbide by surface oxygen termination. 2D Mater. **6**(4), 045025 (2019). https://doi.org/10.1088/2053-1583/ab30f9
